# Deep Learning and Explainable Artificial Intelligence for Infrared-Thermography-Based CMT-Status Classification in Dairy Cows: A Comparative Evaluation in Egyptian Farms

**DOI:** 10.3390/vetsci13101031

**Published:** 2026-09-29

**Authors:** Alaa T. Elmaria, Elsayed Badr, Sobhy M. A. Sallam, Marwa F. A. Attia, Sara Swedian

**Affiliations:** 1 Department Artificial Intelligence, Faculty of Computers and Artificial Intelligence, Benha University, Benha 19111, Egypt; alaa.taha@ecu.edu.eg (A.T.E.); sweidan_ds@fci.bu.edu.eg (S.S.); 2 Department of Scientific Computing, Faculty of Computers and Artificial Intelligence, Benha University, Benha 19111, Egypt; alsayed.badr@fci.bu.edu.eg; 3 Department of Computer Science, Faculty of Computers and Artificial Intelligence, Misr University for Science and Technology (MUST), Giza P.O. Box 77, Egypt; 4 The Egyptian School of Data Science (ESDS), Benha, Egypt; 5 Department of Animal and Poultry Production, College of Agriculture and Food, Qassim University, Buraydah 51452, Saudi Arabia; 6 Animal Production Research Institute, Agricultural Research Center, Giza 12618, Egypt; marwa.elfeky@alexu.edu.eg

**Keywords:** mastitis, somatic cell count, deep learning, convolutional neural networks, vision transformer, DenseNet, Grad-CAM, explainable AI, dairy cattle, infrared thermography

## Abstract

Early and accurate identification of mastitis-associated inflammation is critical to increasing animal comfort, reducing financial losses, and enhancing milk quality. Although infrared thermography (IRT) has emerged as a viable non-invasive screening technique, the relative efficacy of convolutional neural networks (CNNs) and Vision Transformers (ViTs) for automated CMT-status classification from thermal udder pictures is still unknown. This work carefully analyzed seven ImageNet-pretrained deep learning architectures using 976 thermal udder images (708 healthy and 268 mastitic images) from 488 Holstein cows (354 healthy cows, 708 images; 134 mastitic cows, 268 images), including two Vision Transformer models (ViT-B/16 and Swin-Tiny) and five CNN models (ResNet-50, DenseNet-121, EfficientNet-B0, MobileNetV2, Inception-V3). Before training, pictures were preprocessed using contrast-limited adaptive histogram equalization (CLAHE), scaled to 224 × 224 pixels, and divided using cow-oriented grouping intended to reduce animal-level data leakage (this grouping could not be independently verified against a ground-truth cow roster; see Limitations). Each model was developed from start to finish and evaluated using an independent hold-out test set and five-fold animal-level cross-validation. DenseNet-121 had the greatest results on the hold-out test set, with an accuracy of 82.2%, an AUC of 0.922, a sensitivity of 90.0%, and a specificity of 79.2%. Additionally, it achieved the highest cross-validation performance (mean AUC = 0.914 ± 0.019). According to statistical analysis, DenseNet-121 was statistically indistinguishable from Inception-V3 under both tests and from ResNet-50 under the more conservative corrected resampled *t*-test, but significantly outperformed EfficientNet-B0, MobileNetV2, and both Vision Transformers (*p* < 0.05), while all CNN models outperformed both Vision Transformer models (*p* < 0.05). Explainable artificial intelligence (XAI) investigation utilizing Grad-CAM corroborated the biological plausibility of the learnt plausibility characteristics by showing that heat patterns in the udder region had a significant impact on model predictions. Grad-CAM analysis showed that heat patterns in the udder region partly drove model predictions, though attention was occasionally influenced by background regions. The best-performing model was tested without retraining on an independent external cohort of 85 cows from a different farm. Although external performance decreased (accuracy = 60.0%; Cohen’s *κ* = 0.199), consistent with the expected effects of domain shift, the somatic cell count was significantly higher in CMT-positive cows (*p* < 0.001; AUC = 0.783), supporting the biological significance of the identified thermal abnormalities. Fine-tuned CNNs, led by DenseNet-121, delivered accurate and reproducible thermal CMT-status classification under internal cross-validation, but transportability to an independent farm was limited (external accuracy = 0.600, *κ* = 0.199), indicating that domain adaptation and multi-farm validation are needed before broader deployment. These findings provide strong support for the application of CNN-based deep learning in IRT-assisted CMT-status screening while highlighting the necessity for larger multicenter datasets to further evaluate transformer-based approaches. aptation and multi-farm validation are needed before broader deployment. These findings provide strong support for the application of CNN-based deep learning in IRT-assisted mastitis screening while highlighting the necessity for larger multicenter datasets to further evaluate transformer-based approaches.

## Simple Summary

Mastitis reduces milk output and quality while harming animal comfort. It is one of the most expensive diseases in dairy farming. If caught early and without invasive treatments, farmers can respond more quickly. To find out if modern deep-learning models can directly detect mastitis from infrared thermal images of the udder, we looked at two types of models: traditional convolutional neural networks and the latest Vision Transformers. Using the same set of thermal images as in our previous research, we trained seven end-to-end models and tested them with an animal-level cross-validation method designed to prevent data leakage between cows. The convolutional models, especially DenseNet-121, performed significantly better than the Vision Transformers on this relatively small dataset. We also explored the connection between model predictions and somatic cell count, using an explainability method to show where each model focused. Our results suggest that advanced convolutional networks are currently the most reliable option for thermal mastitis screening. However, vision transformers likely need much larger datasets to compete.

## 1. Introduction

A serious and common pathological illness that has a big financial impact on dairy cattle populations is bovine mastitis. In addition to endangering animal welfare and the long-term sustainability of dairy operations, its consequences include reduced milk output and quality, increased veterinary costs, early animal culling, and significant financial harm [1,2,3,4].

Early detection is essential because inflammatory markers often appear prior to overt clinical signs, allowing the disease to spread covertly throughout a herd of cattle. Conventional diagnostic techniques, such as bacterial culture, somatic cell counting (SCC), the California Mastitis Test (CMT), polymerase chain reaction (PCR), and electrical conductance tests, continue to be the accepted standards for clinical evaluation [5,6]. However, these methods are not feasible for continuous, group-wide surveillance within contemporary precision dairy management paradigms because they need laboratory intervention, milk purchase, specialist human resources, or lengthy analytical periods [1,2,3,4].

These indicators differ in what they measure: CMT, SCC, and electrical conductivity are indirect screening indicators of inflammation, whereas bacterial culture and PCR identify the causative intramammary infection itself. CMT—the reference standard used throughout this study—falls into the former category and reflects an inflammatory response rather than a confirmed microbiological diagnosis.

Due to its ability to identify higher under surface temperatures that indicate inflammatory reactions before overt clinical signs appear, infrared thermography (IRT) has emerged as a favorable, non-invasive technique for early mastitis screening [7,8,9,10,11]. Unlike traditional diagnostic methods, IRT allows for quick, non-contact, and instantaneous assessment without upsetting the animals. However, thermal photography is highly sensitive to environmental factors, animal movement, physiological differences, and imaging conditions, making visual interpretation alone insufficient for reliable diagnosis. As a result, using artificial intelligence to power automated picture analytics has become essential for extracting clinically relevant features from thermal data [9,10].

Innovative studies used manually designed temperature and textural characteristics to combine IRT with conventional machine-learning techniques. In addition to highlighting the limitations of manual feature generation and the decreased transferability across various agricultural settings and imaging environments, this research showed the feasibility of automated CMT-status classification [11,12,13,14]. This conceptual framework has been completely reconfigured by the emergence of deep learning, which enables end-to-end learning directly from thermal images, eliminating the need for manually created features and continuously improving diagnostic efficacy [15,16,17,18,19,20,21,22,23,24]. By first identifying the appropriate anatomical regions before extracting thermographic data relevant to categorization, several recent studies have advanced automated thermal analysis. A deep-learning architecture that independently identified the udder regions from thermal pictures was introduced by Zhang et al. [15], However, later studies included heat data from both anatomical sites using YOLO-based localization and segmentation networks, resulting in improved mastitis detection capabilities [16,17,18]. On large thermal imaging archives, directly applied Convolutional Neural Network (CNN) techniques have also demonstrated higher classification precision [19]. Despite these advancements, most previous studies evaluated only one deep-learning architecture, relied on image-centric validation that might inflate performance metrics, or lacked interpretability and rigorous statistical analyses [1,15,16,17,18,19,20,21,22,23,24,25,26,27].

Notably, the majority of thermal-IRT and machine-learning mastitis studies reviewed here (and summarized comparatively in Section 4.1)) report image-, herd-, or record-level validation rather than verified animal-level validation. When multiple images or records from the same animal can appear in both training and test partitions, reported performance can be inflated through pseudoreplication.

The current study aims to further improve diagnostic accuracy using end-to-end deep learning, building on our previous research [1], which demonstrated the feasibility of identifying bovine mastitis using infrared udder thermography through deep features derived from a pre-trained EfficientNetB3 model coupled with conventional machine learning classifiers. While the previous framework produced encouraging results, it was limited by its reliance on static feature extraction and lacked extensive statistical comparisons, comprehensive model fine-tuning, the use of Vision Transformer architectures, methods for model interpretability, and evaluation on an external validation. Preprocessing and architecture choices in the present study were not informed by the pilot study’s results. To overcome these limitations, this study fine-tunes two Vision Transformer models (ViT-B/16 and Swin-Tiny) and five advanced convolutional neural networks (ResNet-50, DenseNet-121, EfficientNet-B0, MobileNetV2, and Inception-V3), all of which are assessed under the same experimental conditions. Additionally, the methodology incorporates external validation on a different cohort, uses Grad-CAM for model explainability, incorporates group-aware validation intended to reduce data leaking (Section 2.3), and performs thorough statistical analyses, including a conservative design-effect correction for possible residual within-cow clustering (Section 2.10).

These modifications are intended to determine the best robust deep learning paradigm for the detection of bovine mastitis using infrared thermography and to offer a comprehensive and reliable evaluation of algorithmic performance [1,25,26,27,28,29,30].

This study aims to identify the most robust deep-learning architecture for cow-level CMT-status classification based on infrared thermography by directly comparing traditional CNNs and Vision Transformers in a standardized evaluative framework. It also addresses significant shortcomings of previous studies, including reliance on static feature extraction, limited architectural comparisons, poor interpretability, and subpar validation techniques.

## 2. Materials and Methods

### 2.1. Dataset and Image Acquisition

**Figure 1:**
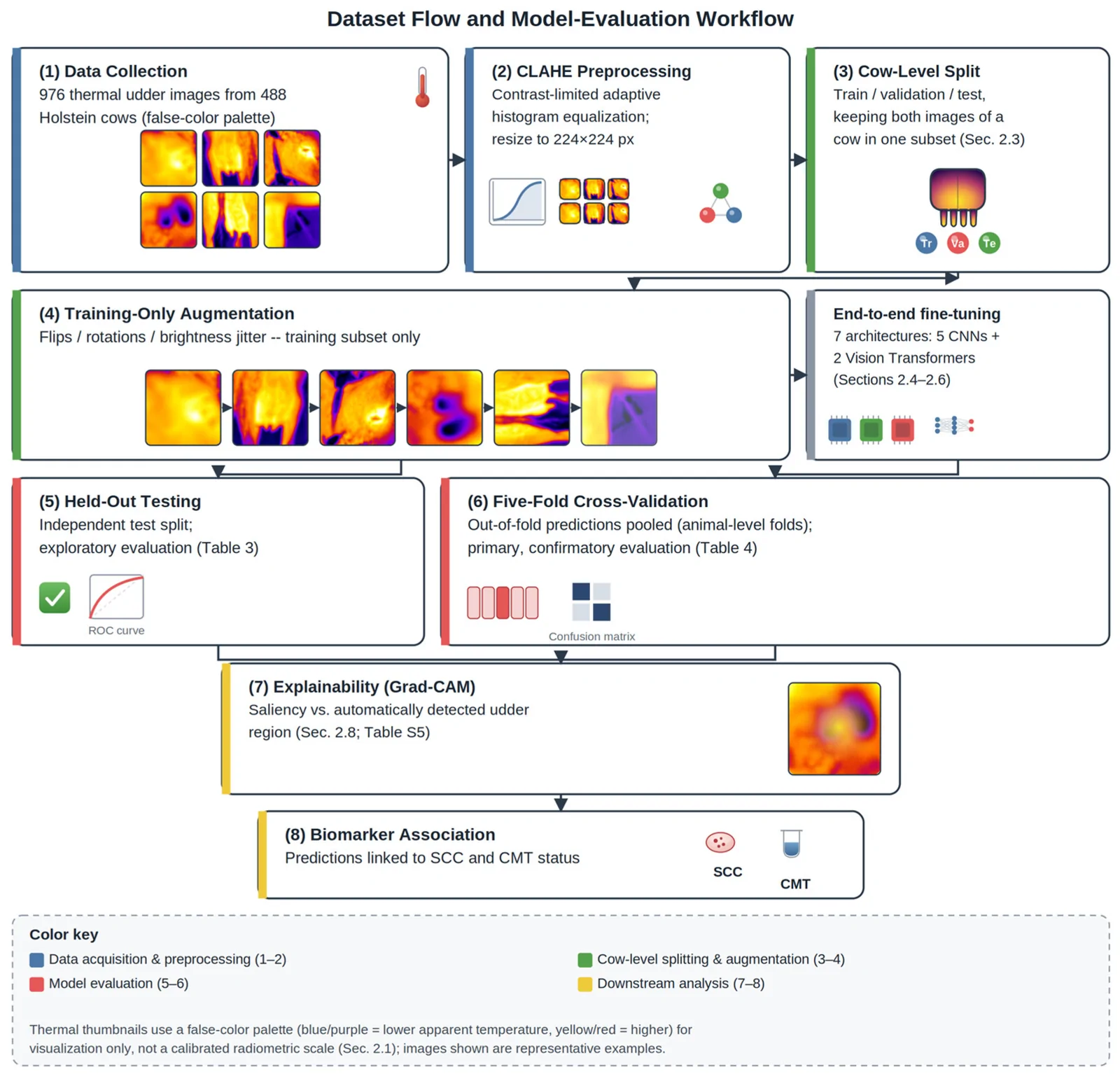
Data collection, CLAHE preprocessing, cow-level splitting prior to augmentation, training-only augmentation, held-out testing, five-fold animal-level cross-validation, explainability, and biomarker association comprise the dataset flow and model-evaluation procedure.

Data from a total of 488 Holstein dairy cows were used to compile the dataset, yielding 976 infrared thermal udder images—708 classified as healthy and 268 as mastitic. The photos used in our companion study were taken prior to milking [1], allowing for a direct methodological comparison between the end-to-end deep learning strategy assessed here and the frozen-feature machine learning pipeline provided there. Each animal in the companion research underwent the California Mastitis Test (CMT), which was used to give class designations at the cow/whole-udder level [1]: CMT-positive cows were labeled as mastitic, whereas CMT-negative cows were labeled as healthy. Images of a particular cow’s left and right udder sides shared the same cow-level classification. Clinical and subclinical instances were not distinguished, and quarter-level diagnostic was not carried out. To make the current analysis self-contained, this label definition is reiterated below. The external validation cohort discussed in Section 2.9, whose cow-side CMT readings were recorded separately at the time that cohort was collected, was then subjected to the same CMT-based labelling protocol independently. Table 1 summarizes the preprocessing parameters and dataset features, and Figure 1 shows the entire data-to-evaluation workflow.

Table S1 (Supplementary Materials) reports the technical specifications of the thermal camera used for image acquisition (UNI-T UTx313; Uni-Trend Technology (China) Co., Ltd., Dongguan, China; uncooled vanadium-oxide microbolometer; 384 × 288 IR resolution; 8–14 µm spectral range; 12 µm pixel pitch; NETD ≤ 40 mK). Beyond the camera model itself, several acquisition parameters recommended for radiometric thermography were not recorded for this archive and could not be reconstructed retrospectively: emissivity setting, exact image distance and angle, ambient temperature and humidity, time relative to milking, udder cleaning/drying protocol, post-handling acclimatization time, and operator consistency across sessions are not available. Given these gaps, the thermal inputs should be understood as exploratory classification of rendered-palette images rather than calibrated radiometric measurements, consistent with the construct-validity caveats noted below.

The bitmap (BMP) format was used to store the thermal images. A fixed preprocessing pipeline (Section 2.2) was used to normalize contrast and spatial resolution prior to model input since thermal cameras encode temperature as a false-color or grayscale map and because the acquisition distance and framing varied somewhat between animals. The mapping from color to absolute temperature is dependent on the camera’s palette-scaling behavior because the images were the camera’s generated BMP outputs rather than raw radiometric temperature matrices. We conservatively assume that the palette may have been auto-scaled per capture (min–max within each frame) because it was not possible to confirm that the device exported a fixed, absolute palette range. Under this assumption, brightness is comparable within an image but not necessarily across images, and any learned signal should be interpreted as a relative, per-image thermal pattern rather than a calibrated absolute-temperature measurement. Fixed-range radiometric export and coupled ambient-condition logging are advised for subsequent acquisitions because there is a real construct-validity concern (Section 4.2). The dataset’s modest imbalance (around 2.6 healthy images for every mastitic image) was specifically addressed during training using class weighting and during evaluation using threshold-independent metrics like AUC (Section 2.11).

### 2.2. Image Preprocessing

After converting each image to the LAB color space, the luminance (L) channel was subjected to Contrast-Limited Adaptive Histogram Equalization (CLAHE) [31] to improve local thermal contrast while reducing noise amplification. The image was then converted back to RGB. With an 8 × 8 tile grid and a clip limit of 2.0, CLAHE was applied identically to all images (healthy and mastitic) using these fixed parameters, determined before any labels were used, so it cannot introduce class-discriminating artifacts by construction.CLAHE enhances the visibility of local temperature gradients on the udder without excessively enhancing sensor noise. Images were scaled prior to CLAHE and standardized to 224 × 224 pixels, the native input size needed by both Vision Transformers, in order to limit memory consumption and guarantee that contrast enhancement worked at a consistent scale. To match the distribution predicted by the pretrained backbones, pixel intensities were normalized using ImageNet channel statistics (mean [0.485, 0.456, 0.406], standard deviation [0.229, 0.224, 0.225]). Only the training subset was subjected to data augmentation, which included horizontal flipping, rotation up to ±20°, random resized cropping (scale 0.8–1.0), and minor color jitter (brightness ±0.1, contrast ±0.1, saturation ±0.1). The validation and test fractions were left untouched so that evaluation represented unaltered photos.

### 2.3. Image-Level Data Partitioning

An image-level split would enable photos of a single cow to appear in both training and test subgroups, inflating performance through pseudo replication, since several thermal images may come from the same animal. To reduce this risk, partitioning was performed using a per-image identifier extracted from the source archive and intended to represent the source animal: whole identifier-groups—never individual photos—were assigned to the training, validation, and test subsets, and the same grouping constraint was used in cross-validation (Section 2.7). This identifier could not be independently verified against a ground-truth cow roster, because no acquisition logbook or metadata file mapping images to individual animals was available for this archive (Section 4.2). The partitioning therefore cannot be certified as strictly cow-disjoint, and results based on it should be interpreted as nominally, rather than verified, animal-level. The quantitative impact of possible residual within-cow clustering on our statistical conclusions is addressed directly through a conservative design-effect correction (Section 2.10).

### 2.4. Deep-Learning Architectures

**Figure 2:**
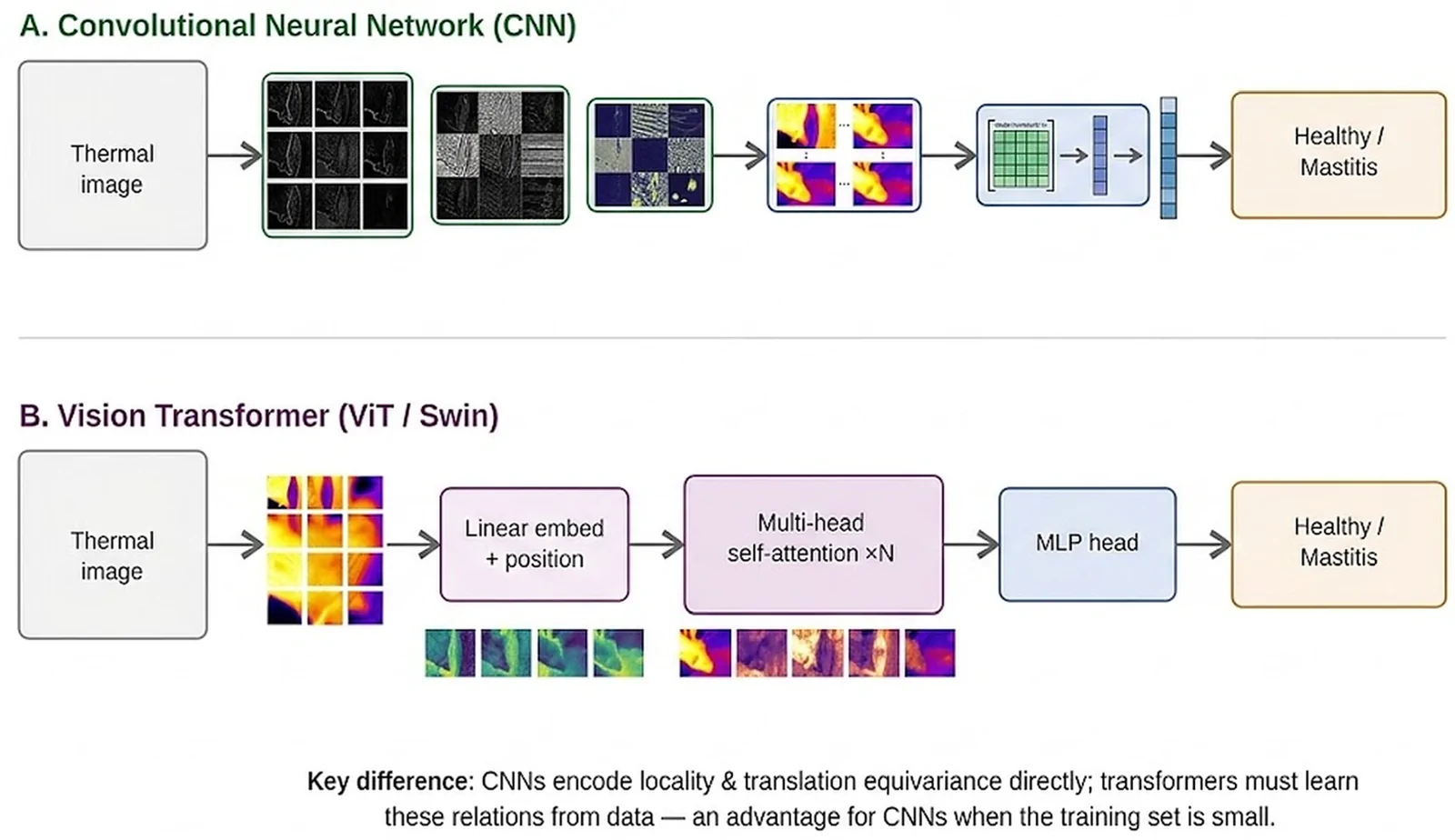
The two families of architecture are compared schematically. (**A**) A convolutional network uses layered convolutions to extract hierarchical local features, then a fully connected head and global pooling. (**B**) A Vision Transformer divides the image into patches, incorporates positional data into them, and uses multi-head self-attention to model global relationships.

Five CNNs and two Vision Transformers were among the seven ImageNet-pretrained architectures that were assessed. They were all instantiated using the timm library and refined end-to-end using a new two-class classification head (Table 2). Under identical data, preprocessing, optimization, and evaluation settings, this design isolates the impact of architectural family. The two transformers represent the canonical global-attention design (ViT-B/16) and the hierarchical shifted-window design (Swin-Tiny), while the five CNNs represent a variety of design philosophies: residual learning (ResNet-50), dense feature reuse (DenseNet-121), compound scaling (EfficientNet-B0), lightweight depth wise-separable convolutions (MobileNetV2), and multi-scale factorized convolutions (Inception-V3). Figure 2 summarizes the conceptual differences between the two families: transformers divide the image into patches and learn spatial relationships through self-attention, which usually requires more training data, while CNNs directly encode locality and translation equivariance through convolution.

Architectural formulation. Each output of the convolutional models reacts to a local receptive field that reflects translation equivariance and locality. This is accomplished by convolving a learnt kernel K over the input feature map I. DenseNet-121 maximizes feature reuse and gradient flow by connecting each layer to all preceding layers within a block; ResNet-50 adds identity skip links to facilitate deep stack optimization; MobileNetV2 uses inverted residuals with linear bottlenecks and depthwise-separable convolutions, Inception-V3 factorizes convolutions across parallel multi-scale branches, and EfficientNet-B0 employs mobile inverted-bottleneck (MBConv) blocks with squeeze-and-excitation under a compound depth/width/resolution scaling rule.

Vision Transformers. The 224 × 224 input is initially divided into non-overlapping patches (16 × 16 for ViT-B/16, yielding *N* = *HW*/*P*^2^ = 196 tokens) by both transformers. Each patch is then flattened and linearly projected to an embedding, a learnable class token is prepended, and positional embeddings are added. Global relationships are then modeled by a stack of transformer encoder layers using multi-head self-attention (MSA), where each token attends to every other token [25,32]. Swin-Tiny decreases the quadratic cost to near-linear and reintroduces a mild locality prior by substituting hierarchical shifted-window attention for global attention and limiting self-attention to local windows that shift across levels [26].

Training algorithm. The exact process below was used to fine-tune each of the seven networks from beginning to end, with the exception of the architecture. (1) Attach a new two-unit classification head and initialize the backbone with ImageNet-pretrained weights. (2) Calculate logits and a class-weighted cross-entropy loss with label smoothing (0.05) for every mini-batch of CLAHE-enhanced, augmented, 224 × 224 images. (3) Using a cosine-annealing schedule and mixed precision, update all parameters using AdamW (base learning rate 3 × 10^−4^, weight decay 1 × 10^−4^). (4) After every epoch, assess the validation AUC; if it does not improve after six epochs (or for the fixed epoch budget in cross-validation), keep the best checkpoint and quit early. (5) Score the unaltered test fold and restore the optimal checkpoint. Within each cross-validation fold, the same loop was run on a training partition that did not include any photos of the fold’s test cows.

### 2.5. Thermal Appearance of Healthy and Mastitic Udders

**Figure 3:**
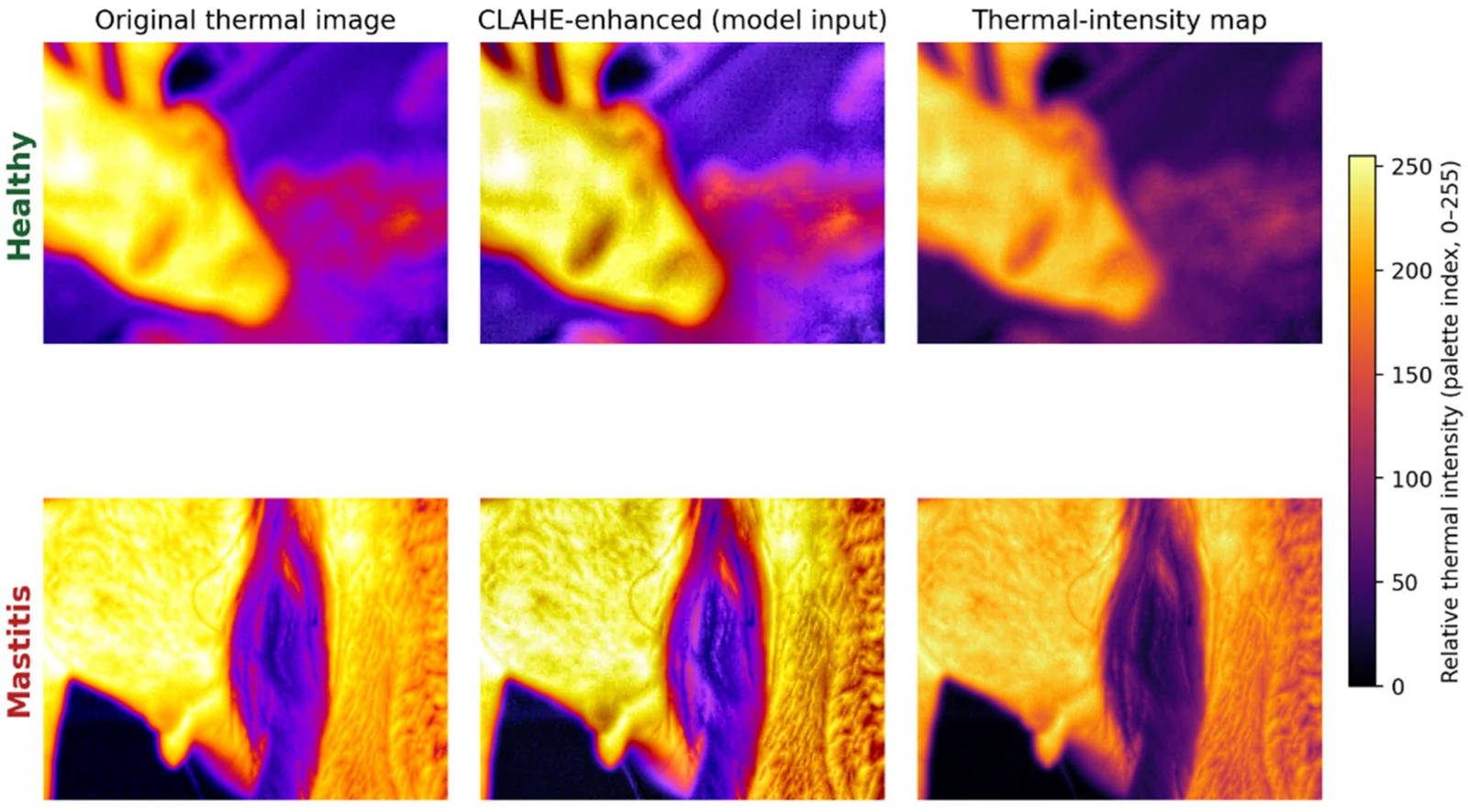
Typical thermal udder pictures. Healthy in the top row, mastitic in the bottom. Columns include the original thermal image, the model input augmented by CLAHE, and the relative thermal-intensity map (palette index, 0–255). The mastitic udder in this typical pair exhibits a wider warm zone; nevertheless, this is not a dataset-wide assertion but rather an informative single-case comparison (see Section 2.5).

**Figure 4:**
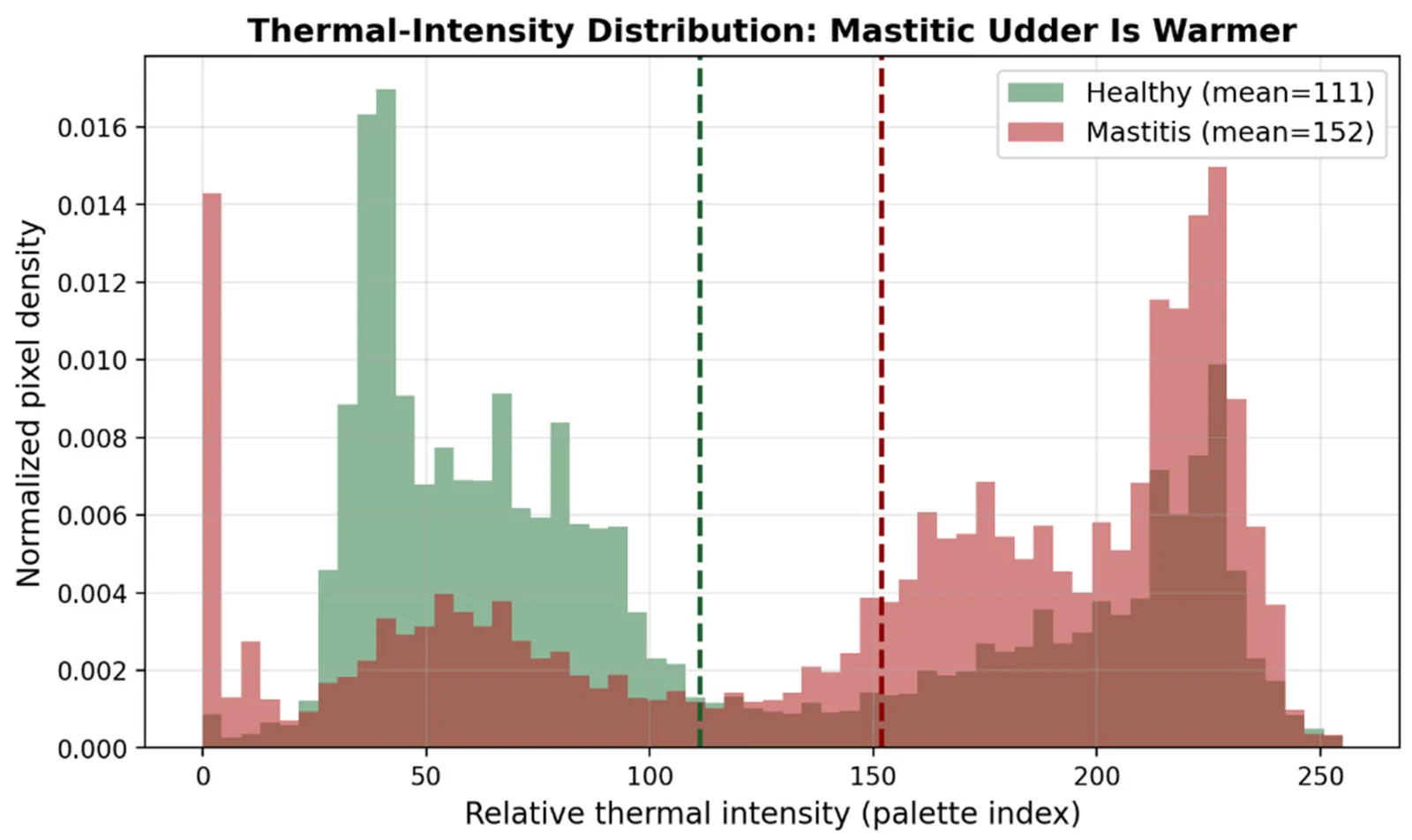
The two typical photos’ respective thermal intensity distribution (palette index). The mastitic image has a greater percentage of high-intensity pixels and is skewed toward higher intensities (mean ≈ 152 vs. ≈111).

Figure 3 shows a comparison of representative healthy and mastitic thermal pictures, and Figure 4 shows the pixel-intensity distributions of these images to show the biological signal that the models use. The palette index offers a monotonic proxy for relative surface temperature because the photos are saved in palette format (darker = cooler, brighter = warmer). With a greater mean thermal intensity and a higher percentage of high-intensity pixels, the mastitic udder in these illustrative specimens is both statistically and obviously warmer than the healthy udder, which is consistent with the local hyperthermia predicted by mammary inflammation. The whole-frame intensities also include non-udder background, thus they are not calibrated temperatures; these panels are illustrative of individual cases rather than a dataset-wide statistical claim.

### 2.6. Training Configuration

**Figure 5:**
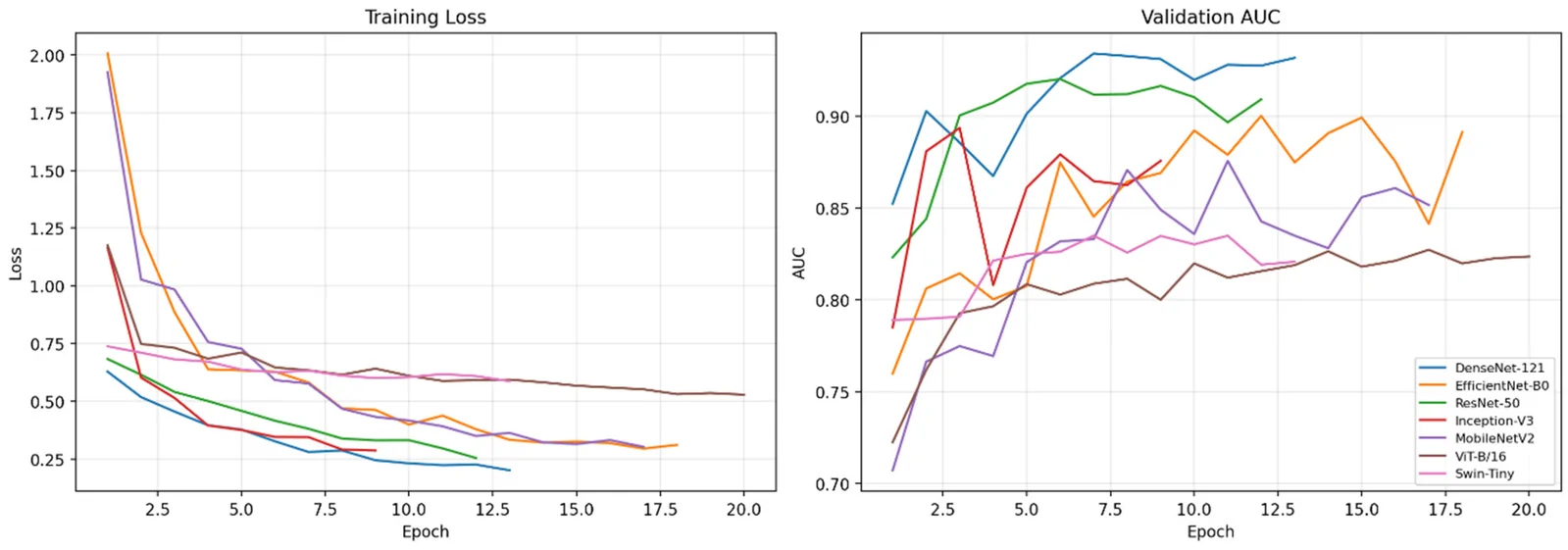
Training loss (**left**) and validation AUC (**right**) for each of the seven designs over time. Convolutional networks outperform Vision Transformers in terms of convergence speed and validation AUC.

All models were trained using a cosine-annealing learning-rate schedule for up to 20 epochs and the AdamW optimizer [33] (initial learning rate 3 × 10^−4^, weight decay 1 × 10^−4^). Label smoothing (0.05) and class-weighted cross-entropy were used to resolve class imbalance. For efficiency, mixed-precision training was employed. The checkpoint with the greatest validation AUC was kept for testing after early stopping was applied to the validation AUC with a patience of six epochs. All architectures used the same hyperparameters and a fixed random seed (42) to guarantee comparability. On a single NVIDIA T4 GPU (NVIDIA Corporation, Santa Clara, CA, USA) via Google Colab (Google LLC, Mountain View, CA, USA), experiments were conducted in PyTorch 2.11.0 (PyTorch Foundation, San Francisco, CA, USA) using the timm 1.0.27 model library (Hugging Face, New York, NY, USA).; the batch size was 16, and augmentation included rotation (±20°), horizontal flip, resize to 256 × 256, random scaled crop to 224 × 224 (scale 0.8–1.0), and minor color jitter. The data-availability repository contains the training scripts and exact library versions. The two transformers converged to consistently lower validation AUC, but training loss continuously fell while validation AUC plateaued for the top CNNs after about 10 epochs (Figure 5).

### 2.7. Animal-Level Cross-Validation

Five-fold stratified group cross-validation was used, stratifying by class and grouping by the per-image identifier described in Section 2.3, so that no identifier-group appeared in more than one fold, with the goal of approximating an animal-level estimate of generalization (subject to the verification limits described in Section 2.3 and Section 4.2). A group-aware validation split (GroupShuffleSplit, stratified by class and grouped by cow, fixed random seed) comprising 15% of each outer training fold was set aside for early stopping, regenerated independently within each of the five outer folds so that no cow appears in both the inner validation slice and the corresponding outer test fold., and out-of-fold predictions were aggregated for each model so that each image was predicted exactly once by a fold that had never seen the animal. A single fixed cow-level train/validation/test split is used in the held-out assessment (Section 3.1) and the cross-validation (Section 3.2), which are two evaluations of the same 976-image cohort under different partitioning techniques (Table 1), while the latter examines each image exactly once by repartitioning all animals across five folds. The two Vision Transformers were evaluated on the held-out test set first due to their significantly lower held-out performance and significantly higher computational cost; their cross-validation was then carried out using the same technique.

More generally, across the full model set, the fixed held-out test evaluation (Section 3.1) was executed before the five-fold cross-validation (Section 3.2) in the recorded analysis pipeline. No hyperparameters, architectural choices, class thresholds, or the composition of the model set were altered after the held-out test results were inspected; the model highlighted for downstream explainability and external-cohort analyses was selected using the cross-validated (out-of-fold) ranking rather than the single held-out split. Nonetheless, because the held-out results were viewed prior to cross-validation, we treat the single-split held-out evaluation (Section 3.1) as exploratory and the five-fold cross-validation (Section 3.2) as the primary, confirmatory estimate of generalization, consistent with its use throughout the Results and Discussion.

### 2.8. Model Explainability (Grad-CAM)

The best-performing model was subjected to gradient-weighted class activation mapping (Grad-CAM), which produced heatmaps that show the image regions most important to the mastitis prediction. The target layer for convolutional networks was the last convolutional layer. Since Grad-CAM was only applied to the chosen best model (DenseNet-121), a transformer-specific variant that reshapes token activations to a spatial grid was created for completeness but is not reported here. As a result, no transformer attention maps are shown. To determine if discriminative attention was focused on udder heat areas rather than background or acquisition artifacts, representative healthy and mastitic test pictures were seen.

### 2.9. External Validation Cohort and Association with Reference Tests

After the original study was finished, an independent external validation cohort was formed to evaluate generalization beyond the development data. 85 Holstein cows from a different commercial dairy farm were included in this cohort. They were photographed at a different acquisition period using the same thermal-imaging and preprocessing methodology as outlined in Section 2.2 and Section 2.3. There was no cow-identifier overlap with the 488 animals in the primary dataset, and none of these animals or their thermal images were utilized at any phase of model building, including training, hyperparameter selection, validation, or internal cross-validation. A contemporaneous cow-side California Mastitis Test (CMT), somatic cell count (SCC), and panel of blood and milk biomarkers were recorded for each cow as the reference standard. After applying the final trained DenseNet-121 model to these images without retraining or fine-tuning, the accuracy, sensitivity, specificity, and Cohen’s *κ* of its predictions were assessed against the CMT reference.

Using techniques suitable for the data, the association between somatic cell count (SCC) and mastitis state was honestly investigated. A non-parametric Mann–Whitney U test was used to compare SCC between groups, along with the discrimination (AUC) of raw SCC and logistic regression on log10-transformed SCC, because SCC distributions differ significantly between healthy and affected cows and can cause quasi-complete separation in logistic models. Convergence problems caused by separation were stated as such; no *p*-values were imputed or modified toward significance.

Without retraining or fine-tuning, the final trained DenseNet-121 model was applied to the independent external validation cohort mentioned above to create the deep-learning label used in the SCC and CMT analyses (Section 3.5 and Section 3.6). These estimates are truly out-of-sample and represent an independent external evaluation rather than an in-sample agreement analysis because that cohort’s thermal images are stored under their own sample-level index and do not share a cow identifier with the primary 976-image set. The observed external concordance with the CMT reference is a direct indicator of transportability to a new location because construction eliminates any optimism from data overlap. The preserved multilayer-perceptron outputs from the companion study serve as the previous machine-learning predictions that were compared [1], applied without retraining to the identical images. The Mann–Whitney U test was employed for between-group SCC comparisons [34], and Cohen’s *κ* was utilized for agreement between categorical predictions [35]. The two analyses use slightly different subsets of the external cohort: all cows with a paired cow-side CMT record (*n* = 85) are used in the CMT-based evaluation (Section 3.6), while cows with an entirely missing SCC value are excluded from the SCC analyses (Section 3.5), leaving *n* = 77, whereas cows whose SCC was recorded only as a non-numeric bound (such as “<90,000” or “>9,000,000”) are retained using conservative numeric substitutions (90,000 and 9,000,000 cells/mL, respectively).

### 2.10. Statistical Comparison of Models

The aggregated out-of-fold predictions were subjected to pairwise comparisons in order to see whether architectural differences were statistically significant or merely coincidental. Because all models were evaluated on the same pictures, differences between ROC curves were analyzed using the DeLong test for two correlated AUCs. Furthermore, fold-wise AUCs were compared with the Nadeau–Bengio corrected resampled *t*-test, which is more conservative than a naïve paired *t*-test since it accounts for the reliance between overlapping cross-validation training sets. The significance level was set at *p* < 0.05. The six DeLong *p*-values from this family were adjusted for multiplicity using the Holm–Bonferroni step-down procedure [30] because the primary architecture comparison was pre-specified as DenseNet-121 (the best model) against each of the other six architectures. Both raw and adjusted values are reported.

A further limitation affects the precision of these tests: a verified per-image cow identifier could not be reconstructed from the source image archive, so the train/validation/test partitioning and five-fold cross-validation could not be certified as cow-disjoint, and images from the same cow (nominally paired left- and right-side captures) may be treated as independent observations by the tests above. To avoid overstating precision under this residual pseudoreplication, we additionally applied a conservative design-effect correction (DEFF = 1 + (m − 1) × ICC, with the known fixed cluster size m = 2 images per cow) to widen confidence intervals and deflate test statistics, evaluated across a sensitivity range of intraclass correlation (ICC = 0.3–0.7) representative of paired anatomical thermal measurements. Comparisons that remained significant across this entire range are reported as robust to within-cow clustering; comparisons that lost significance under this correction are reported as such (Section 3.3; Supplementary Table S3).

### 2.11. Evaluation Metrics

Accuracy, area under the ROC curve (AUC), sensitivity (recall for the mastitis class), specificity, precision (positive predictive value), and F1-score were used to report performance; mastitis was considered the positive class. For every model, ROC curves and confusion matrices were produced.

*Precision* = *TP*/(*TP* + *FP*)

*Accuracy* = (*TP* + *TN*)/(*TP* + *TN* + *FP* + *FN*)

*Sensitivity* (*Recall*) = *TP*/(*TP* + *FN*)

*Specificity* = *TN*/(*TN* + *FP*)

F1 = 2 (*Precision* × *Sensitivity*)/(*Precision* + *Sensitivity*)

Because of the class imbalance, the main model-selection criterion was the area under the receiver-operating-characteristic curve (AUC), which aggregates discrimination across all thresholds independently of any single operating point. Cohen’s *κ* was used to quantify chance-corrected concordance between two categorical labelings for the agreement analysis [35],

*κ* = (*p_o_*− *p_e_*)/(1 − *p_e_*)

where *p*_o_ is the observed agreement and *pₑ* is the chance-expected agreement. Cross-validated metrics are presented as mean ± standard deviation across folds, and uncertainty on the held-out point estimations was measured using 1000-resample bootstrap 95% confidence intervals. Each design is given a constant color that is utilized consistently in all multi-model figures to facilitate visual tracking between figures.

## 3. Results

Because a verified cow identifier could not be reconstructed for this archive (Section 2.3), the point estimates and intervals reported in Table 3, Table 4 and Table 5 below treat each image as the unit of analysis and should not be read as if the underlying observations were independent cows. We therefore treat the design-effect-corrected intervals and test statistics in Section 2.10 and Section 3.3, and Supplementary Table S3—which explicitly account for the residual within-cow clustering—as the primary basis for judging which findings are statistically robust; the tables below are retained in their naive, unadjusted form for completeness and for direct comparability with the previous pipeline [1]. Readers evaluating the strength of any single comparison should consult the corrected figures in Section 3.3 and Section 4.2 rather than the raw intervals alone.

### 3.1. Held-Out Test Performance

DenseNet-121 performed the best overall on the held-out test set (accuracy = 0.822, AUC = 0.922, sensitivity = 0.900, specificity = 0.792, *F*1 = 0.735), closely followed by ResNet-50 (AUC = 0.879) and EfficientNet-B0 (AUC = 0.912). ViT-B/16 and Swin-Tiny only achieved AUCs of 0.725 and 0.716, respectively, while all five CNNs significantly beat both Vision Transformers (Table 3). For a screening application, where missing an afflicted animal is more expensive than a false alarm, the top CNNs’ high sensitivity is ideal.

**Table 3:** The held-out test findings for the seven architectures (mastitis = positive class). The models are arranged using AUC. Table 4 offers the more trustworthy multi-fold estimates, while the values are point estimates from a single training run at a fixed seed on a nominally cow-level split (Section 2.3); each measure has naive bootstrap 95% confidence intervals, which do not account for possible within-cow clustering—see Supplementary Table S3 for the design-effect-corrected intervals we treat as primary (Section 2.10). which do not account for possible within-cow clustering—see Supplementary Table S3 for the design-effect-corrected intervals we treat as primary (Section 2.10).

Model	Accuracy	AUC	Sensitivity	Specificity	F1	PPV	NPV
**DenseNet-121**	0.822 (0.760–0.884)	0.922 (0.875–0.961)	0.900 (0.806–0.977)	0.792 (0.709–0.870)	0.735 (0.626–0.821)	0.621	0.955
**EfficientNet-B0**	0.808 (0.740–0.863)	0.912 (0.859–0.954)	0.925 (0.825–1.000)	0.764 (0.682–0.838)	0.725 (0.619–0.817)	0.597	0.964
**ResNet-50**	0.740 (0.671–0.808)	0.879 (0.819–0.934)	0.900 (0.800–0.980)	0.679 (0.593–0.762)	0.655 (0.547–0.750)	0.514	0.947
**Inception-V3**	0.781 (0.706–0.843)	0.860 (0.794–0.913)	0.725 (0.574–0.861)	0.802 (0.720–0.873)	0.644 (0.512–0.748)	0.580	0.885
**MobileNetV2**	0.726 (0.651–0.801)	0.858 (0.791–0.921)	0.825 (0.711–0.943)	0.689 (0.596–0.775)	0.623 (0.510–0.732)	0.500	0.913
**ViT-B/16**	0.651 (0.575–0.733)	0.725 (0.637–0.809)	0.650 (0.500–0.795)	0.651 (0.557–0.740)	0.505 (0.384–0.619)	0.413	0.831
**Swin-Tiny**	0.637 (0.562–0.706)	0.716 (0.630–0.793)	0.725 (0.581–0.857)	0.604 (0.505–0.686)	0.523 (0.400–0.625)	0.408	0.853

**Figure 6:**
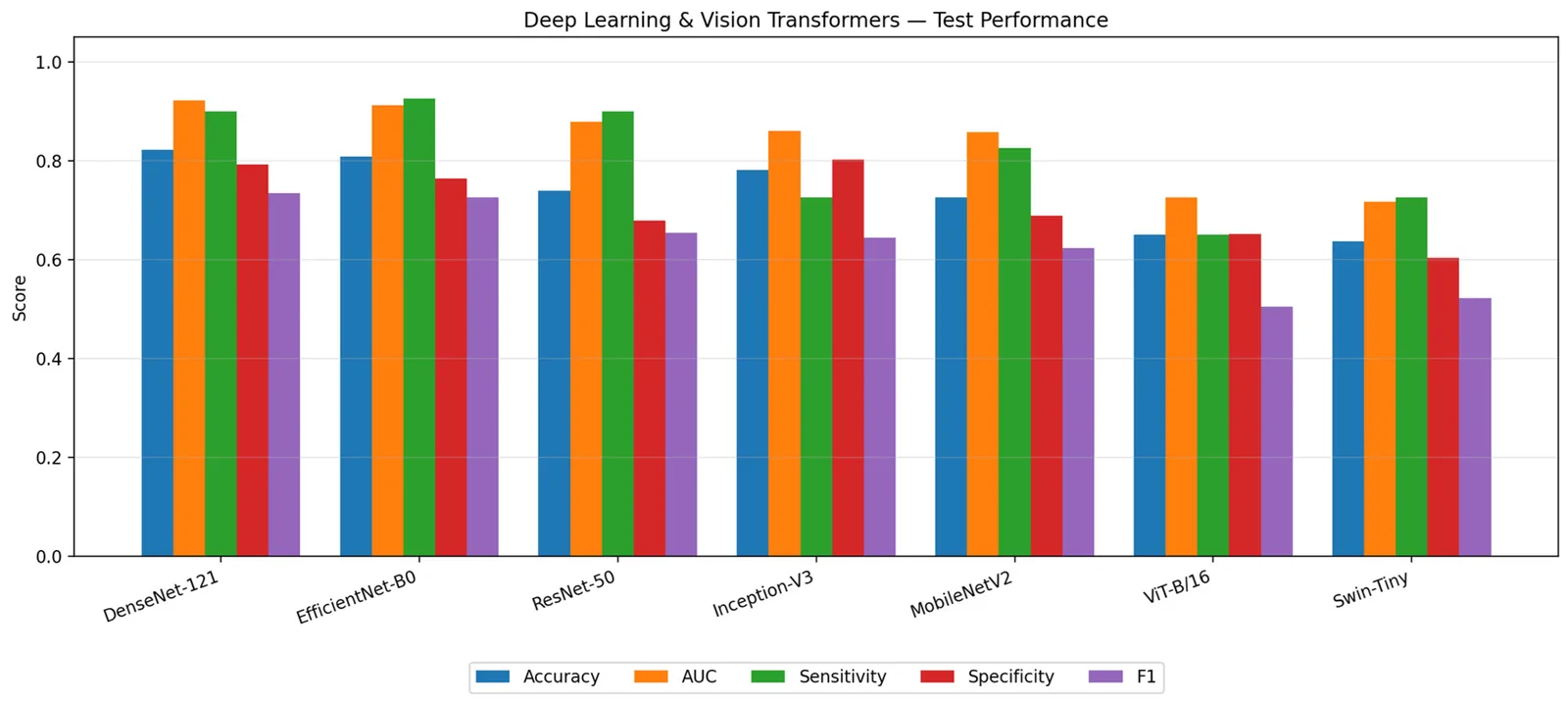
The accuracy, AUC, sensitivity, specificity, and *F*1 of the seven architectures were held out for testing.

**Figure 7:**
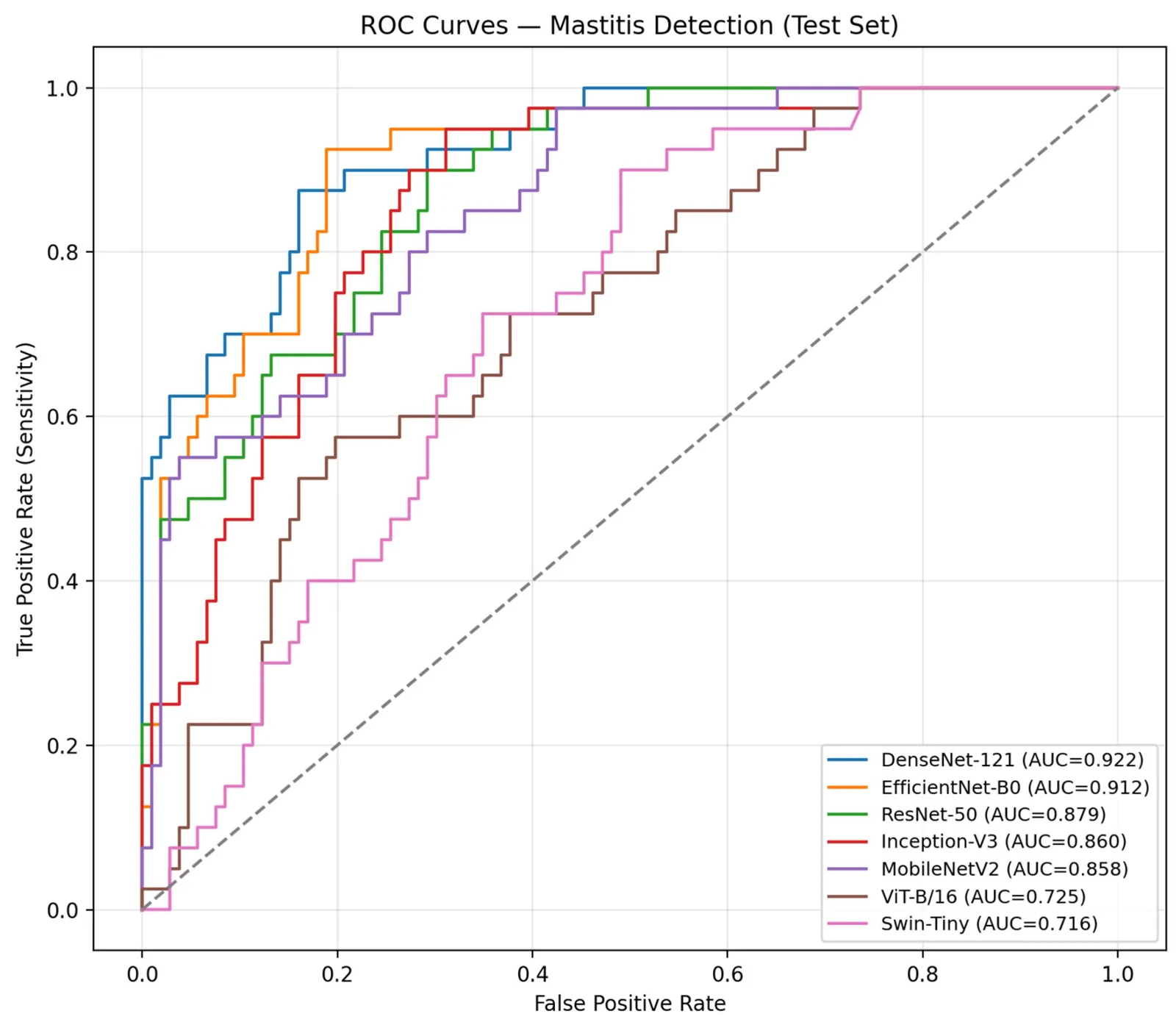
The held-out test set’s receiver-operating-characteristic (ROC) curves. The two Vision Transformers are obviously subordinated to the five CNNs.

**Figure 8:**
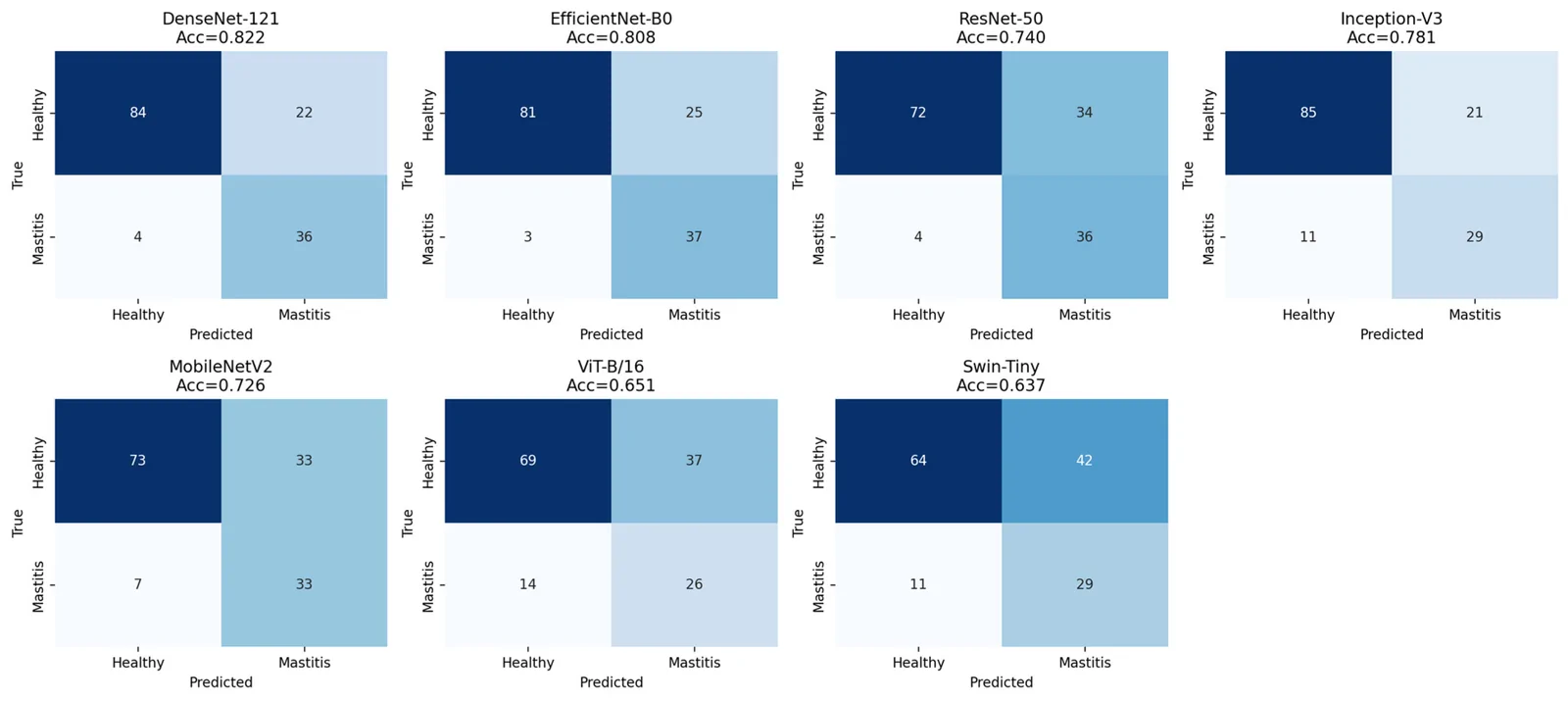
Confusion matrices for each of the seven architectures on the held-out test set.

The receiver-operating-characteristic curves and the per-metric comparison (Figure 6 and Figure 7) also show the same ordering, with the CNN curves falling significantly above the transformers’ curves over the whole operating point range. Figure 8 displays per-model confusion matrices on the held-out set. Figure 6 provides precision–recall curves for all architectures, which replicate the same CNN-over-transformer ordering and are more informative than ROC under class imbalance [36]. Table 3’s point estimates, particularly for the seed-sensitive transformers, should be viewed in conjunction with the cross-validated results in Table 4 and the bootstrap confidence intervals since it represents a single training run at a fixed seed on a single split.

**Table 4:** All seven structures underwent five-fold stratified group cross-validation (nominal animal-level grouping; Section 2.3) (mean ± standard deviation). The reported standard deviations capture fold-to-fold variability only; they are not adjusted for possible within-cow clustering, which is addressed separately through the design-effect correction in Section 2.10 and Section 4.2.

Model	Accuracy	AUC	Sensitivity	Specificity	Precision	F1
**DenseNet-121**	0.835 ± 0.018	0.914 ± 0.019	0.825 ± 0.040	0.839 ± 0.038	0.664 ± 0.042	0.734 ± 0.015
**Inception-V3**	0.823 ± 0.019	0.903 ± 0.018	0.746 ± 0.048	0.852 ± 0.018	0.656 ± 0.034	0.698 ± 0.035
**ResNet-50**	0.794 ± 0.051	0.890 ± 0.026	0.843 ± 0.048	0.775 ± 0.085	0.599 ± 0.066	0.696 ± 0.038
**EfficientNet-B0**	0.764 ± 0.035	0.842 ± 0.034	0.754 ± 0.047	0.768 ± 0.058	0.558 ± 0.046	0.639 ± 0.029
**MobileNetV2**	0.704 ± 0.047	0.814 ± 0.029	0.787 ± 0.019	0.672 ± 0.065	0.481 ± 0.051	0.596 ± 0.040
**ViT-B/16**	0.676 ± 0.132	0.768 ± 0.033	0.477 ± 0.344 (median 0.574 [IQR 0.189–0.604])	0.751 ± 0.289	0.457 ± 0.147	0.389 ± 0.204 (median 0.486 [IQR 0.290–0.539])
**Swin-Tiny**	0.622 ± 0.173	0.727 ± 0.056	0.468 ± 0.404 (median 0.585 [IQR 0.000–0.755])	0.680 ± 0.363	0.236 ± 0.206	0.303 ± 0.254 (median 0.435 [IQR 0.000–0.473])

### 3.2. Animal-Level Cross-Validation Performance

The held-out ranking was replicated using five-fold cow-level cross-validation, which also yielded a more conservative, repeatable estimate for each of the seven architectures. The two Vision Transformers came in last (ViT-B/16 AUC = 0.768 ± 0.033; Swin-Tiny AUC = 0.727 ± 0.056); DenseNet-121 had the highest mean AUC (0.914 ± 0.019) and mean accuracy (0.835 ± 0.018). Inception-V3 (AUC = 0.903 ± 0.018) and ResNet-50 (AUC = 0.890 ± 0.026). While the transformers were significantly more variable among folds, the CNNs had minor standard deviations (Section 3.3). These differences were formally tested (Section 3.3) rather than presumed since the margins separating the top CNNs are similar to their fold-to-fold variability. Interestingly, compared to the frozen-feature machine-learning pipeline reported on the same data (mean AUC 0.681 ± 0.132), the cross-validated AUC of DenseNet-121 (0.914 ± 0.019) is significantly more stable [1], highlighting the advantages of end-to-end fine-tuning for out-of-fold generalization.

**Figure 9:**
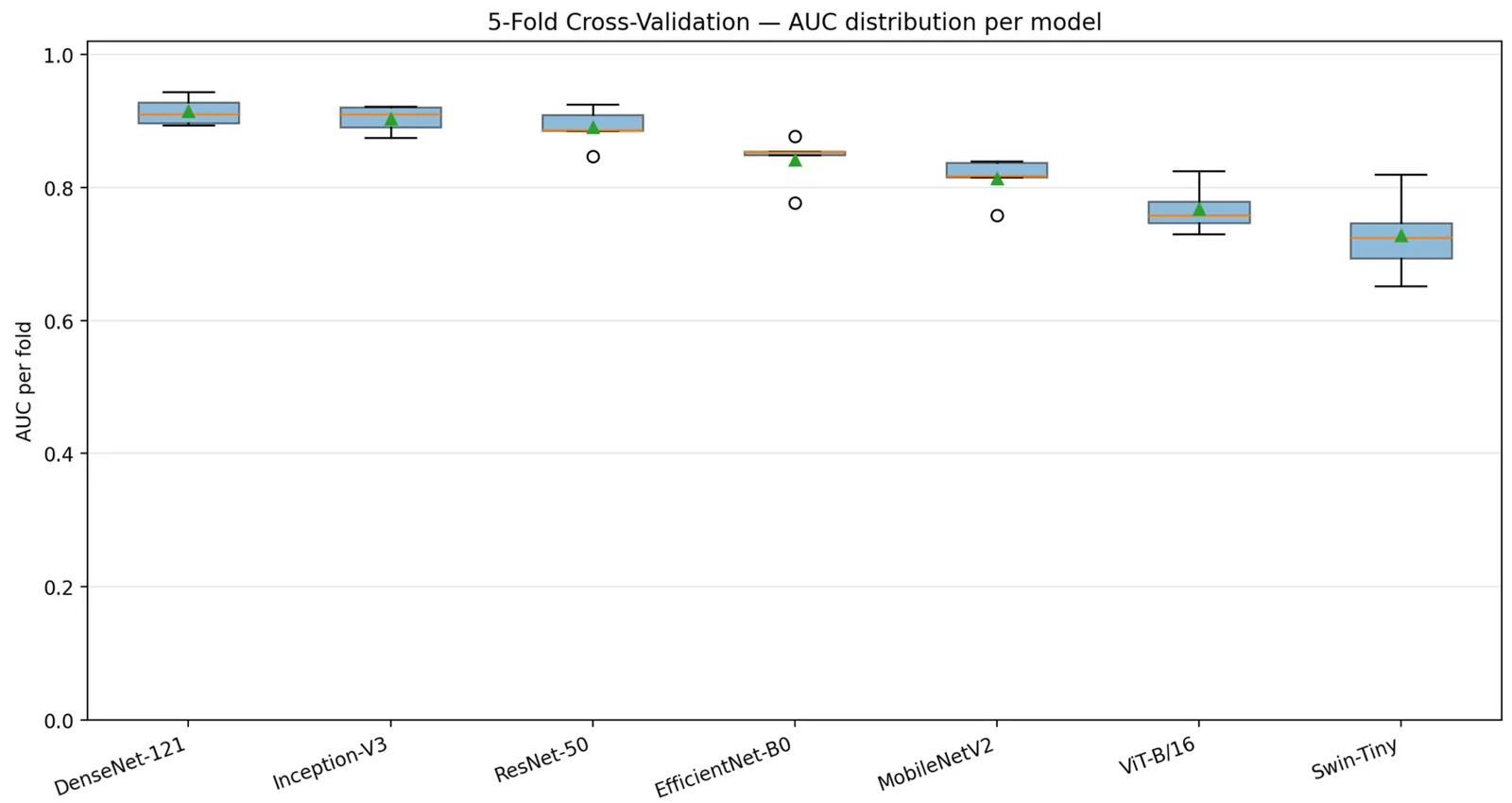
All seven architectures’ per-fold AUC distribution during five-fold animal-level cross-validation. Boxes span the interquartile range (25th–75th percentile), the horizontal line marks the median, whiskers indicate the range of non-outlier values, circles denote outlier folds, and the triangle marks the mean. Compared to the CNNs, the two transformers have a noticeably broader dispersion.

**Figure 10:**
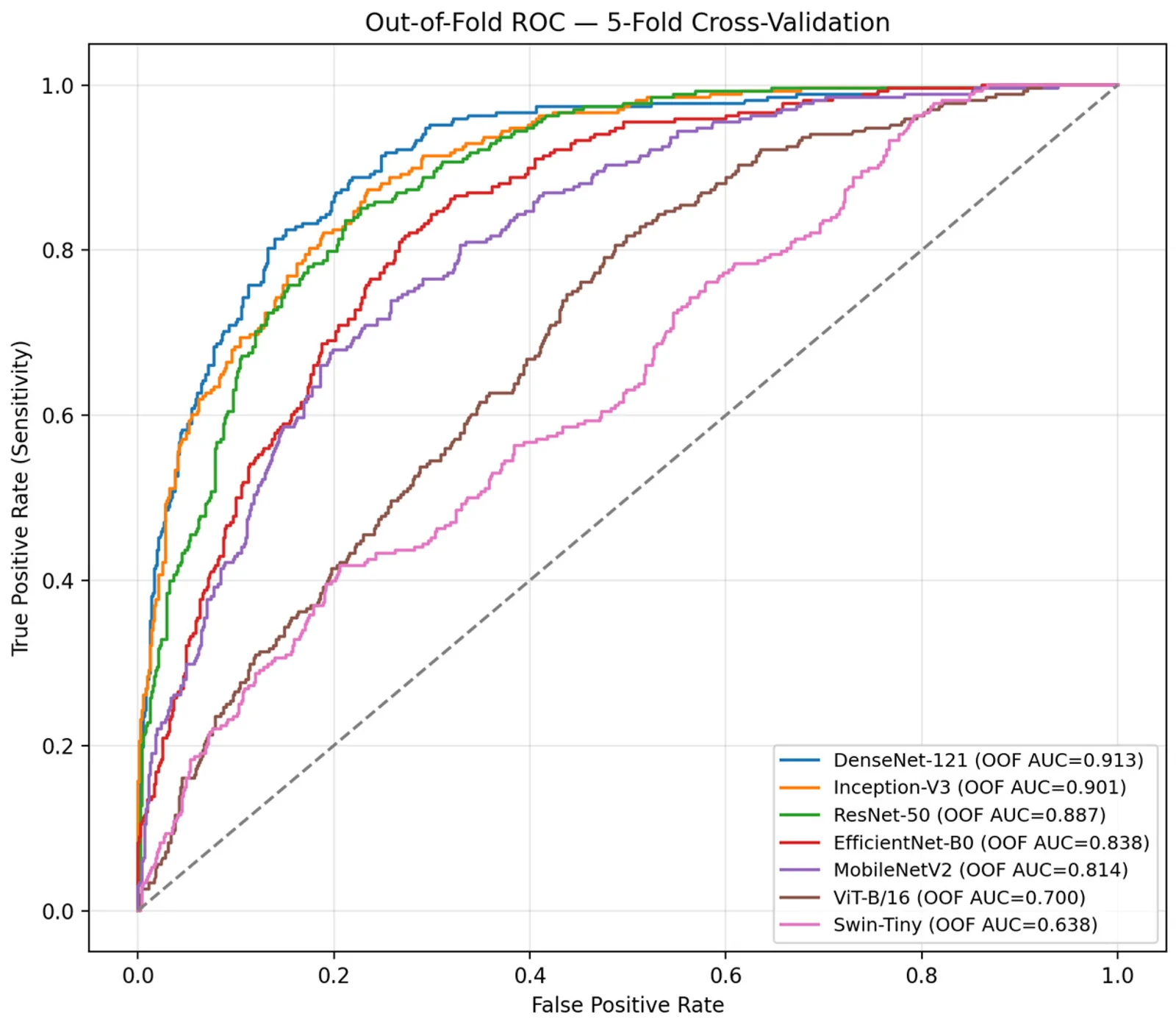
Out-of-fold ROC curves from five-fold cross-validation; a fold blind to each image’s animal.

**Figure 11:**
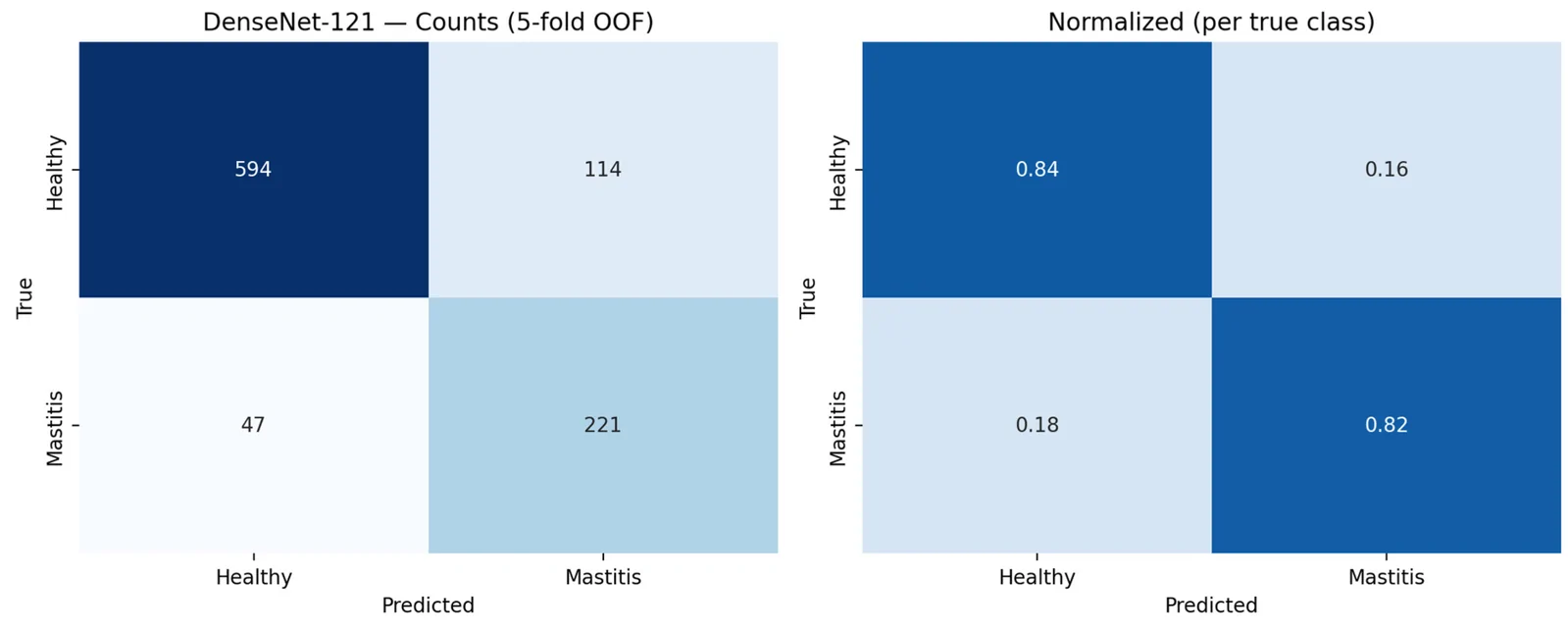
DenseNet-121 out-of-fold confusion matrix aggregated across five folds (counts and per-class normalized values).

According to the per-fold AUC distributions (Figure 9), DenseNet-121 exhibits stable behavior across folds by combining the tightest spread with the highest median AUC. The combined out-of-fold ROC curves (Figure 10) validate the ranking and the out-of-fold confusion matrix for DenseNet-121 by predicting each image exactly once by a fold that had not been trained on the same grouped identifier (Figure 11) shows balanced errors across classes.

The held-out discovery was validated and refined through cross-validation of the two Vision Transformers. The transformers were significantly unstable across folds in addition to their lower mean AUC (ViT-B/16, 0.768 ± 0.033; Swin-Tiny, 0.727 ± 0.056). Their sensitivity varied greatly (ViT-B/16, 0.477 ± 0.344; Swin-Tiny, 0.468 ± 0.404), and their F1-scores collapsed on some folds (Swin-Tiny F1, 0.303 ± 0.254). In contrast, all CNNs maintained low standard deviations for every statistic. The practically significant finding is this instability, not just a lower average: transformer performance cannot be relied upon fold by fold on a dataset this size. The mean ± SD reported for these transformer distributions in Table 4 should only be interpreted as a coarse summary because they are strongly non-normal and near-collapse in some folds; Figure 9 displays the full per-fold values, and a median [*IQR*] summary would more accurately represent these specific rows (e.g., Swin-Tiny precision and *F*1) than mean ± SD. (Because pooling reveals this fold-to-fold variability, the pooled out-of-fold AUCs in Figure 10, 0.700 for ViT-B/16 and 0.638 for Swin-Tiny, are lower than the per-fold means in Table 4).

According to the DenseNet-121 aggregated out-of-fold confusion matrix (Table 5), 594 out of 708 healthy images were correctly classified (specificity ≈ 0.84), whereas 221 out of 268 mastitic images were accurately detected (sensitivity ≈ 0.82) and 47 were missed. Table 6 provides the corresponding per-class report. Given that a “healthy” output is accurate in the vast majority of cases, the strong negative predictive value is especially essential for screening.

**Table 5:** DenseNet-121’s aggregate out-of-fold confusion matrix (all 976 pictures, five-fold cross-validation).

Actual\Predicted	Healthy	Mastitis	Total
**Healthy**	594 (TN)	114 (FP)	708
**Mastitis**	47 (FN)	221 (TP)	268
**Total**	641	335	976

**Table 6:** DenseNet-121’s performance on out-of-fold predictions by class.

Class	Precision	Recall	F1-Score	Support
**Healthy**	0.927	0.839	0.881	708
**Mastitis**	0.660	0.825	0.733	268
**Macro avg**	0.793	0.832	0.807	976

### 3.3. Statistical Comparison and CNN-Versus-Transformer Results

Which differences are significant was made clear by pairwise significance testing on the aggregated out-of-fold predictions (Table 7). DenseNet-121 significantly outperformed ResNet-50 under the DeLong test (Holm-adjusted *p* = 0.033), although this difference was not significant under the more conservative corrected resampled *t*-test (Holm-adjusted *p* = 0.116). By the DeLong test, DenseNet-121 and Inception-V3 were statistically indistinguishable (pooled out-of-fold ΔAUC = 0.012, Holm-adjusted DeLong *p* = 0.265), forming a joint top tier. The top three CNNs (DenseNet-121, Inception-V3, and ResNet-50) were comparable under the more cautious Nadeau–Bengio corrected resampled *t*-test, but DenseNet-121 still performed much better than EfficientNet-B0, MobileNetV2, and both transformers. Importantly, every CNN performed considerably better than both Vision Transformers in both tests (all *p* < 0.05), although the two transformers themselves varied, with ViT-B/16 slightly but significantly outperforming Swin-Tiny (pooled out-of-fold AUC 0.700 vs. 0.638; DeLong *p* = 0.003). Because DenseNet-121 (with Inception-V3 as a statistical equal) leads and the CNN-over-transformer advantage is statistically established rather than incidental, the practical conclusion is robust to test selection.

**Table 7:** DenseNet-121’s pairwise relevance on out-of-fold predictions compared to the other designs. The corrected resampled *t*-test compares fold-wise AUCs, DeLong tests correlated AUCs, and *ΔAUC* is DenseNet-121 minus the comparator. The DeLong test analyzes *ΔAUC*, which is the pooled out-of-fold difference (DenseNet-121 minus the comparator); the two *p*-value columns are Holm–Bonferroni-adjusted across the six pre-specified comparisons (raw values are in the reproducibility output significance_holm.csv). Following correction, DenseNet-121 is statistically indistinguishable from Inception-V3 in both tests and from ResNet-50 in the more conservative corrected resampled *t*-test. It is still significantly superior to EfficientNet-B0, MobileNetV2, and, most importantly, both Vision Transformers in both tests (all Holm-adjusted *p* < 0.05). Therefore, multiplicity correction does not affect the CNN-over-transformer advantage.

Comparison (DenseNet-121…)	ΔAUC	DeLong *p* (Holm)	Corr-t *p* (Holm)	Verdict
**vs. Inception-V3**	+0.012	0.265	0.686	n.s. (comparable)
**vs. ResNet-50**	+0.026	0.033	0.116	Not robust (DeLong sig. only if ICC < 0.5; *t*-test n.s.)
**vs. EfficientNet-B0**	+0.075	<0.001	0.047	significant
**vs. MobileNetV2**	+0.099	<0.001	0.045	significant
**vs. ViT-B/16**	+0.213	<0.001	0.021	significant
**vs. Swin-Tiny**	+0.275	<0.001	0.032	significant

Because a verified cow identifier was not available (Section 2.10), we additionally re-evaluated Table 7 under a conservative design-effect correction for within-cow clustering (ICC = 0.3–0.7; Supplementary Table S3). Every comparison that was significant by the DeLong test remained significant across this entire conservative range, with one exception: the DenseNet-121 vs. ResNet-50 comparison—already non-significant under the corrected resampled *t*-test (Holm-adjusted *p* = 0.116)—also lost DeLong significance once ICC ≥ 0.5 was assumed. The CNN-over-transformer advantage and DenseNet-121’s superiority over EfficientNet-B0 and MobileNetV2 are therefore robust not only to multiplicity correction but also to plausible within-cow clustering; the DenseNet-121 vs. ResNet-50 difference is not, and should be read as an unresolved, statistically fragile comparison.

Convolutional networks consistently performed better than Vision Transformers in both evaluation protocols. The difference between the best CNN (AUC = 0.922) and the best transformer (AUC = 0.725) on the held-out set was roughly 0.19 AUC, a significant difference that is consistent with CNNs’ established data efficiency in comparison to transformers. With less than 700 training images, Vision Transformers were unable to match the CNNs and, as demonstrated above, behaved unstably across folds. They also lack the inherent inductive biases of locality and translation equivariance and typically require very large training corpora or aggressive regularization to reach their potential. Large annotated datasets are hard to come by in thermal veterinary imaging, where our conclusion is immediately applicable.

### 3.4. Explainability (Grad-CAM)

**Figure 12:**
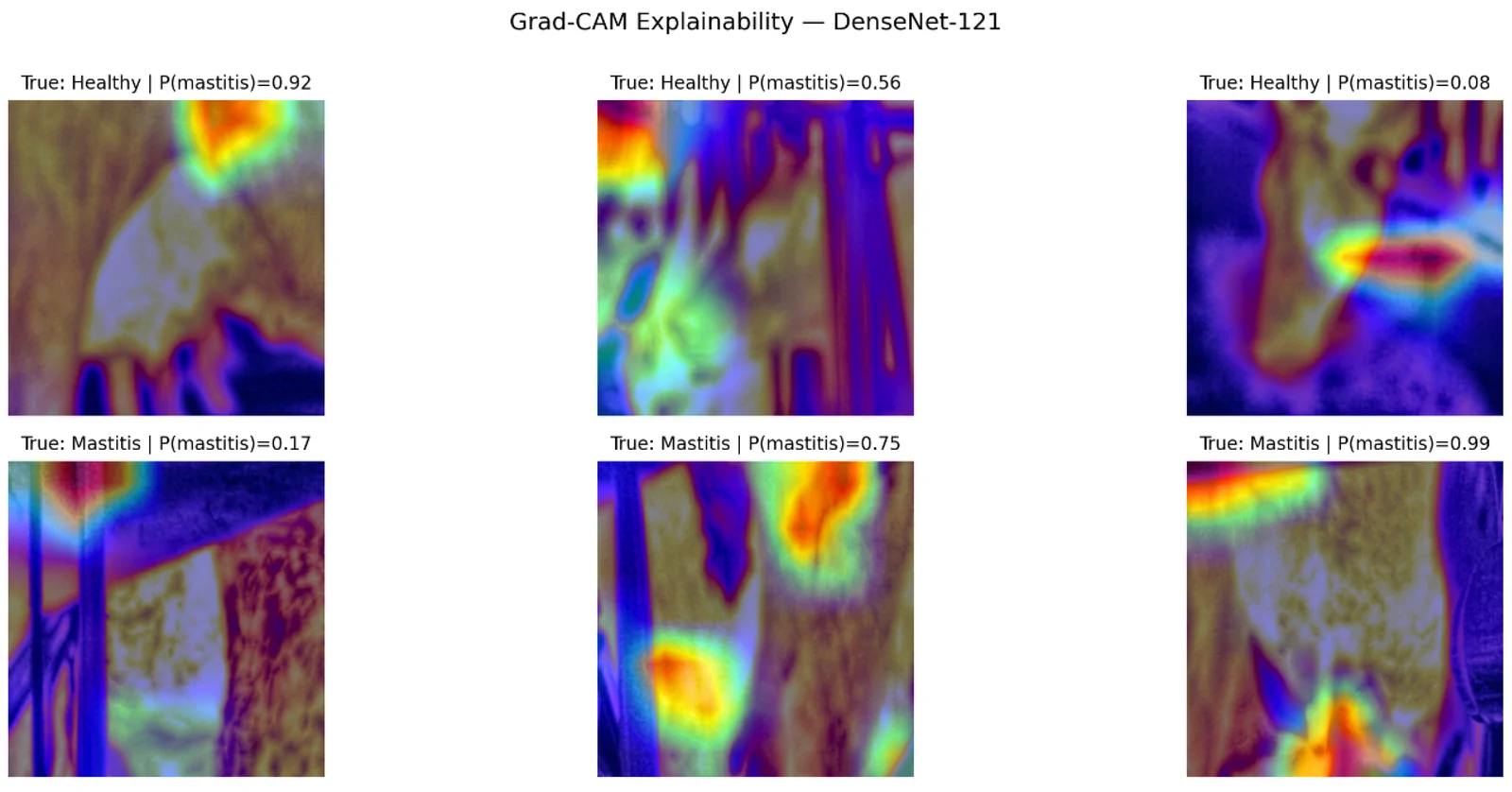
DenseNet-121 Grad-CAM visualizations on one true positive, one true negative, one false positive, and one false negative case, selected by a pre-specified rule. Warm colors show areas that are most important for predicting mastitis; attention is sometimes drawn to the backdrop but frequently correlates with udder thermal regions. In order to avoid the perceptual artifacts associated with rainbow/jet colormaps, heatmaps employ a perceptually homogeneous colormap (magma).

Grad-CAM heatmaps for DenseNet-121 revealed that discriminative attention overlapped with udder thermal regions for many successfully classified instances. For instance, mastitic cases were confidently and correctly identified with attention on warm udder zones (Figure 12). However, attention was partially focused on the background or image peripheral in a subgroup of photos, and several genuinely healthy images had higher probability of mastitis. This behavior suggests that even if the model captures pertinent thermal patterns, it is still not entirely focused on the udder, which encourages future work to explicitly constrain the udder region-of-interest.

### 3.5. Somatic Cell Count Association

Raw SCC distinguished CMT status with an AUC of 0.783, and the somatic cell count was considerably greater in CMT-positive cows than in CMT-negative cows (median 426,500 vs. 140,000 cells/mL; Mann–Whitney U, *p* = 1.0 × 10^−5^) (Table 8). On log10-transformed SCC, logistic regression showed concordance (coefficient = 1.664, *p* = 3.5 × 10^−4^). The thermal-model predictions, on the other hand, did not substantially correlate with SCC (*p* = 0.679; SCC AUC = 0.470), suggesting that cellular inflammation and surface-temperature patterns may represent somewhat complementary elements of udder health rather than a single shared signal.

### 3.6. External Validation on an Independent Cohort

**Figure 13:**
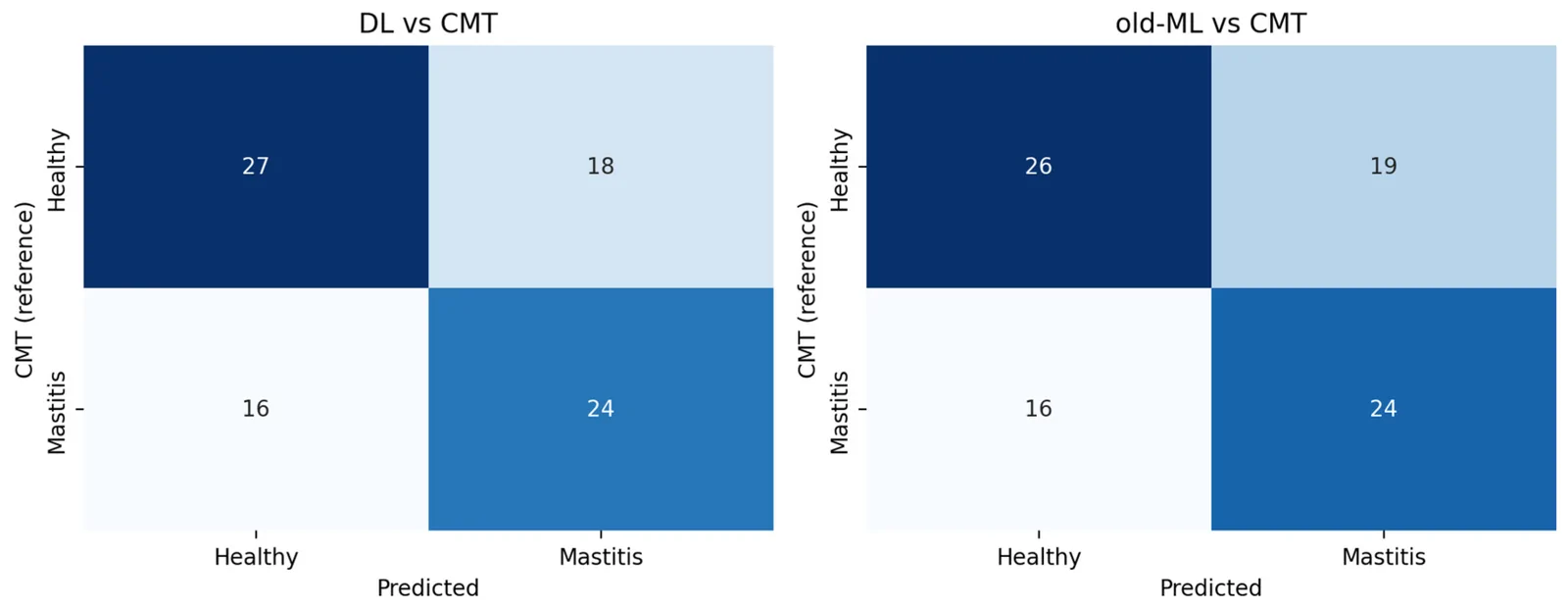
Confusion matrices for external validation comparing the deep learning (**left**) and previous machine learning (**right**) predictions to the California Mastitis Test reference on the independent cohort (*n* = 85).

Using the contemporaneous cow-side California Mastitis Test (CMT) as the reference standard, the final DenseNet-121 model was applied, without retraining, to the independent external cohort of 85 cows (different farm, separate acquisition time; Section 2.9). The model obtained an accuracy of 0.600, sensitivity of 0.600, specificity of 0.600, and Cohen’s *κ* = 0.199 against this external reference (Table 9, Figure 13). Because the categorical metrics above use the fixed 0.5 threshold carried over unchanged from internal validation (no threshold retuning was performed on the external cohort), we additionally report the exact confusion-matrix counts (prevalence 40/85 mastitic, 47.1%: TN = 27, FP = 18, FN = 16, TP = 24) and threshold-free, probability-based metrics computed on the continuous DenseNet-121 output: ROC-AUC = 0.656 (95% bootstrap CI 0.537–0.767), PR-AUC = 0.667 (95% CI 0.526–0.799; baseline PR-AUC at this prevalence = 0.471), PPV = 0.571 (95% CI 0.417–0.721), and NPV = 0.628 (95% CI 0.481–0.769) (Supplementary Table S4). A calibration analysis (logistic recalibration of the continuous score against the CMT outcome) showed poor calibration, with an intercept of −0.184 and a slope of 0.194 (perfect calibration: intercept 0, slope 1), indicating that the model’s predicted probabilities are markedly overconfident on this external cohort relative to observed outcome frequencies (Supplementary Figure S2).

This is a significant drop from the within-cohort cross-validated discrimination (mean AUC 0.914), which is in line with the palette-scaling considerations covered in Section 4.2 and probably represents domain shift related to variations in farm environment and acquisition period. Our companion study’s frozen-feature pipeline [1], applied to the identical external pictures, demonstrated high agreement between the two thermal models (accuracy 0.824, *κ* = 0.647) and comparable external agreement with CMT (*κ* = 0.177). A continuous thermal pattern linked to inflammation that is only partially matched with what CMT measures may be captured by infrared thermography, according to the convergence of two separately generated thermal models on this external cohort and their reduced agreement with CMT. Therefore, the external performance described here needs be validated in larger multi-center cohorts, and thermal screening is best positioned as a supplementary technique alongside proven cow-side diagnostics rather than as a substitute.

## 4. Discussion

In this work, convolutional and transformer topologies for infrared thermography-based CMT-status classification are compared head-to-head for the first time. The main conclusion is clear and statistically supported: fine-tuned CNNs, led by DenseNet-121, greatly outperform Vision Transformers under the limited-data conditions characteristic of veterinary thermal imaging, with each CNN outperforming both transformers under two separate tests. When adapting a pretrained backbone to a tiny, domain-shifted dataset, DenseNet-121’s dense connection facilitates gradient flow and feature reuse. The statistical indistinguishability between DenseNet-121 and Inception-V3 suggests that using a fine-tuned CNN instead of a transformer is more crucial than selecting the strongest CNNs. It is helpful to compare it with our related study. There, an optimistic image-level hold-out AUC was obtained using a frozen EfficientNetB3 feature extractor and classical classifiers, but the cross-validated AUC (0.681 ± 0.132) was significantly lower and more variable [1]. More importantly, end-to-end network tuning reduced fold-to-fold variance by nearly an order of magnitude and raised the cross-validated AUC for DenseNet-121 to 0.914 ± 0.019. This is consistent with letting the network modify its filters to the thermal domain, rather than recycling frozen natural-image characteristics, being an important factor for robust animal-level generalization; however, validation procedure, architecture, statistical methodology, and possibly preprocessing all differ simultaneously between the two studies, so the improvement is associated with, but not uniquely attributable to, end-to-end fine-tuning.

In addition to internal cross-validation, a completely independent external cohort that was later gathered from a different farm was used to assess the model (Section 3.6). The two thermal models continued to agree closely with each other (*κ* = 0.647), but external accuracy dropped to 0.600 and agreement with CMT was weak (*κ* = 0.199) with this real domain shift. When a model trained at one site is exposed to new imaging conditions, it should exhibit this pattern: the underlying thermal signal is internally consistent, but discrimination deteriorates in comparison to the within-cohort estimate. This external test measures the approach’s existing transportability and pinpoints the calibrated, multi-site data collecting required to close the gap prior to deployment, rather than undermining it. This decrease is not surprising because the validation cohort was obtained on a separate farm under different field conditions, which introduced an actual distribution shift that was not taken into account when the model was being developed. Rather of exaggerating the model’s existing transportability, reporting this accurately measures it.

Vision Transformers’ poor performance should not be seen as a flaw in the transformer paradigm as a whole, but rather as a result of the shared training recipe and data scale. It is important to distinguish between two claims: while our evidence indicates that the transformers performed poorly under the same, untuned recipe used here (a single learning rate of 3 × 10^−4^, no warm-up, and 20 epochs shared with the much smaller CNNs), it does not prove that transformers are the incorrect architecture class for thermal udder imagingIn order to keep the comparison under control, a transformer-specific schedule—lower base learning rate, learning-rate warm-up, longer training, and stronger regularization—was purposefully not used here. Transformers require more data to find the hard-coded spatial priors of convolutions since they replace them with learnt attention. In addition to having a lower average, the transformers in our investigation were remarkably unstable between folds, with sensitivity standard deviations greater than 0.34. This meant that a single split may provide an inaccurately optimistic or pessimistic image. When evaluating data-hungry topologies on limited datasets, this instability alone supports the use of animal-level cross-validation instead of single-split evaluation. Our findings draw a realistic line: CNNs are the best option for thermal udder datasets this size, and transformer techniques would probably require significantly larger datasets, transformer-specific pretraining, or hybrid convolutional-attention designs to become competitive.

Clinically speaking, the top models combine moderate precision (about 0.66 at the default operating point) with high sensitivity (0.90) and significant negative predictive value. In practice, the decision threshold can be adjusted to the intended use, trading sensitivity against precision. The precision–recall curves in Supplementary Figure S3, along with performance at a fixed high-sensitivity operating point, further characterize this trade-off. The moderate precision is an expected consequence of class imbalance and generates a non-trivial number of false positives. All things considered, this profile is ideal for a supportive screening role because the system consistently flags the majority of impacted animals and hardly ever misses them, and positive flags require verification by a recognized test like CMT or SCC [37]. It shouldn’t be used as a stand-alone diagnostic substitute.

The models are qualified and validated using the Grad-CAM analysis. The model is partially influenced by non-udder cues, as evidenced by residual attention on background and several confident false positives on healthy images (e.g., predicted mastitis probabilities of 0.81–0.92). However, attention frequently coincided with udder thermal regions, supporting the biological plausibility of the learned features. This is not just a cosmetic constraint, but a possible danger to validity: aggregate measures may be optimistic if some discriminative signal comes from background linked to acquisition rather than actual disease. This worry is lessened by two factors. First, the strong out-of-fold performance was achieved under identifier-based fold separation intended to approximate cow-level separation (Section 2.3); while this reduces the likelihood of trivial identification shortcuts, it cannot be presented as a guarantee given the unverified nature of the grouping identifier, and concentration on udder areas was the predominant trend across successfully categorized cases. Second, we quantified this directly: across all 976 images, a mean of 42.9% (median 41.1%) of each image’s peak Grad-CAM activation (top 20% of activation values) fell outside an automatically detected udder region (Otsu-thresholded, center-weighted largest warm blob, manually spot-checked on multiple random samples; Supplementary Table S5). This fraction was similar for correctly classified (43.1%) and misclassified images (41.1%), suggesting that background-linked attention is a general property of the model’s saliency maps rather than a specific driver of its errors. Because the automatic mask is a coarse intensity-based proxy rather than a verified anatomical segmentation, and because Grad-CAM’s spatial resolution is limited by the final convolutional feature map, this figure should be read as an upper-bound, approximate estimate of background reliance rather than a precise one. Explicit ROI-cropped retraining—rather than post hoc masking of an already-trained model—remains the more direct way to test and potentially reduce this dependence and is identified as a priority for follow-up work.

Lastly, the biomarker analyses provide insight into the meaning of the thermal signal. The biological validity of SCC as an inflammatory measure was confirmed by its substantial and significant elevation in CMT-positive cows, but SCC was not linked to the thermal-model predictions. The deep-learning predictions consistently agreed with the previous machine-learning pipeline (*κ* = 0.647) but only slightly with CMT (*κ* = 0.199): the two thermal models, which were constructed in very different ways, converge on the same signal, but that signal only partially overlaps with the cow-side reference. This consistent pattern implies that surface temperature variations can be measured by infrared thermography without the necessity for one-to-one tracking of cellular counts or CMT scores. However, because they rely on the subset of animals with paired measures (*n* = 77–85) and are hence statistically underpowered, the lesser relationships with SCC and CMT should be regarded with caution. When considered collectively, our findings confirm thermography as a supplementary modality and show promise for multimodal mastitis-risk screening classification when combined with validated assays.

### 4.1. Comparison with the Previous Machine-Learning Pipeline

Due to the fact that this study expands a pipeline that was previously published on the same cohort [1], The contribution of end-to-end deep learning is isolated by a direct comparison (Table 10). The two best models perform similarly on the single image-level held-out split, but under animal-level cross-validation, they diverge significantly. Overall accuracy is slightly lower (0.862 → 0.822) and AUC is comparable (0.918 → 0.922). On the held-out split, the new model trades a little specificity for a large gain in sensitivity (0.741 → 0.900), which is the clinically preferred direction because a missed mastitis case is more costly than a false alarm.

Animal-level five-fold cross-validation shows the most significant difference, with mean AUC increasing from 0.681 ± 0.132 to 0.914 ± 0.019. This difference illustrates the advantage of fine-tuning the convolutional backbone end-to-end rather than freezing it and assigning classification to a shallow model: end-to-end training allows the network to adapt its filters to thermal texture rather than reusing ImageNet-optimized features unaltered. This comparison should not be read as controlling for partitioning strategy: as with the current pipeline (Section 2.3), a verified cow-level partition could not be confirmed for the previous pipeline either, and its split was performed without a recovered animal identifier [38]. Because any residual within-cow clustering is therefore expected to affect both estimates in a similar direction, the AUC gain in Table 10 is best interpreted as a comparison of modeling approach under a shared, unverified partitioning caveat, rather than as evidence that the improvement is attributable to fine-tuning alone. The contribution here is not that deep learning wins on every metric, but rather that it generalizes significantly better to unseen animals, which is the metric that matters for deployment. The previous pipeline retains real advantages, such as lower computational cost, faster training, and slightly higher held-out specificity. Additionally, the current work offers multiplicity-corrected model comparison and Grad-CAM explainability, which were absent from the earlier analysis.

The current investigation is positioned within this literature in Table 11. The majority of thermal studies either stop at temperature thresholds or feed hand-crafted features into classical models; validation is typically reported at the image or record level; models based on conductivity and milk that achieve very high accuracy do so using instrumented-parlour inputs that thermal screening does not require. In contrast to previous thermal-mastitis investigations, the current work fine-tunes both convolutional and transformer backbones end-to-end on the thermal images themselves and validates using group-aware cross-validation intended to approximate animal-level generalization, combined with multiplicity- and clustering-corrected statistical comparison (Section 2.3, Section 2.10 and Section 4.2).

### 4.2. Limitations

These results are tempered by a number of limitations. Most importantly, although partitioning and cross-validation (Section 2.3 and Section 2.7) were designed to group all images from the same cow together, the per-image identifier used for this grouping could not be independently verified against a ground-truth cow roster, because no acquisition logbook or metadata file linking images to individual animals was available for this archive. As a result, the reported cross-validation and held-out results cannot be certified as strictly cow-disjoint and should be interpreted as nominally, rather than verified, animal-level estimates; residual within-cow clustering (each cow contributing a fixed pair of left- and right-side images) may inflate the precision of our estimates relative to a truly independent sample. We address the quantitative consequences of this directly by applying a conservative design-effect correction across a sensitivity range of plausible intraclass correlations (Section 2.10; Supplementary Table S3): the CNN-over-transformer advantage and DenseNet-121’s superiority over EfficientNet-B0 and MobileNetV2 remain significant under the most conservative assumption tested, while the DenseNet-121-versus-ResNet-50 comparison does not and should be treated as unresolved. We regard this as the most consequential limitation of the study and recommend that any future reuse of this dataset prioritize recovering or reconstructing a verified cow identifier. Precision and external validity are constrained by the dataset’s uneven class distribution and small number of farms and animals within a single region. In order to provide a direct test of transportability, model performance was also evaluated on one independent external cohort that was later collected from a different farm (Section 3.6). However, because this cohort was small (*n* = 85) and single center, it establishes external validity for a single new site rather than broad generalizability. Before making any deployment claims, prospective, multi-center external validation spanning more herds, cameras, and acquisition circumstances is still required. The external SCC and CMT studies were based on a small cohort (*n* = 77–85), which restricts the accuracy of the external estimations, and model attention was not yet limited to an udder region of interest. Lastly, the transformers might profit from transformer-specific training recipes not examined here, even though all seven architectures were cross-validated using the same methodology. In a similar vein, all architectures used the same hyperparameter configuration (a single learning rate of 3 × 10^−4^, no warm-up, and 20 epochs) to keep the comparison under control. Since this recipe is not adjusted for the much larger transformers, their weak and unstable results should be interpreted as underperformance under this shared recipe rather than as proof that the transformer class is inherently unsuited to the task. The cross-validated estimates are regarded as primary because the held-out results also come from a single seed and a single split, making them vulnerable to run-to-run volatility. Lastly, a portion of the learned signal may reflect per-image contrast rather than absolute udder temperature because the thermal inputs are rendered BMP palettes rather than calibrated radiometric matrices and the palette may have been auto-scaled per capture. Cow-level CMT labelling, in which both side-images inherit a single cow-level label, can introduce label noise when only one quarter or side is affected; such noise would be expected to bias reported performance downward against the model rather than inflate it, and quarter-level relabeling is identified as a direction for future work. Additionally, because the external cohort was imaged using the same rendered-BMP pipeline, a portion of the external performance drop may reflect cross-site variations in palette scaling and acquisition conditions rather than a failure of the thermal signal itself, which partially explains the modest external DL CMT concordance. Rather than contradicting the main conclusions, these points outline specific next steps.

## 5. Conclusions

DenseNet-121 achieved a cross-validated AUC of 0.914 ± 0.019, demonstrating how fine-tuned convolutional neural networks can accurately, consistently, and comprehensibly classify CMT status from infrared thermal udder images at the image level; because a verified cow identifier could not be recovered, this performance should be interpreted as image-level rather than animal-level (Section 4.2, Limitations). Despite their success on large-scale benchmarks, Vision Transformers performed noticeably worse on this limited-data thermal job, suggesting that CNNs are still the recommended architecture for this application. On the same data, end-to-end fine-tuning significantly increased robustness compared to a frozen-feature pipeline. The model maintained a quantifiable but lower level of performance (accuracy 0.600, *κ* = 0.199) when applied without retraining to an independent external cohort from a different farm. This confirms that larger multi-center validation is still required prior to routine use and quantifies the impact of domain shift. Limiting the model to an udder region of interest, calibrating radiometric acquisition across sites, and integrating thermal imaging with complementary markers are the most promising paths toward practical, on-farm deployment, according to explainability analysis and the somatic-cell-count comparison.

## Figures and Tables

**Table 1:** Features of image datasets and preprocessing settings.

Parameter	Value
Image format	BMP (Bitmap)
Input resolution	224 × 224 pixels
Colour space/enhancement	RGB → LAB (CLAHE on L channel) → RGB
Classes	Healthy, Mastitis
Total images	976 (708 healthy, 268 mastitic)
Cows (animals)	488 Holstein
Split strategy	Grouped by image-derived identifier (nominal cow-level; not independently verified—see Section 2.3)
Train/Validation/Test	684/146/146 images
Class ratio (healthy:mastitis)	≈2.6:1

**Table 2:** Deep-learning architectures evaluated. Parameter counts are approximate.

Architecture	Family	Params (M)	Key Inductive Design
**ResNet-50**	CNN	25.6	Residual skip connections
**DenseNet-121**	CNN	8.0	Dense connectivity, feature reuse
**EfficientNet-B0**	CNN	5.3	Compound depth/width/resolution scaling
**MobileNetV2**	CNN	3.5	Inverted residuals, depthwise separable conv.
**Inception-V3**	CNN	23.8	Multi-scale factorized convolutions
**ViT-B/16**	Transformer	86.6	Global self-attention over 16 × 16 patches
**Swin-Tiny**	Transformer	28.3	Hierarchical shifted-window attention

**Table 8:** Correlation between reference labels and somatic cell count (SCC, cells/mL). Group-specific medians and Mann–Whitney U *p*-values (one-sided, SCC higher in positive class).

Comparison	Median (Neg.)	Median (Pos.)	*p*-Value	SCC AUC
**SCC vs. CMT (reference)**	140,000	426,500	1.0 × 10^−5^	0.783
**SCC vs. DL prediction**	230,500	208,000	0.679	0.470

**Table 9:** Model predictions were externally validated using the California Mastitis Test (CMT) reference on an independent cohort, and there was agreement between the two thermal model generations (*n* = 85).

Comparison	n	Accuracy	Sensitivity	Specificity	Cohen’s *κ*
**DL (DenseNet-121) vs. CMT**	85	0.600	0.600	0.600	0.199
**Prior ML vs. CMT**	85	0.588	0.600	0.578	0.177
**DL vs. prior ML**	85	0.824	0.814	0.833	0.647

**Table 10:** Direct comparison using the same cohort as the prior machine-learning pipeline [1]. Cross-validation values are animal-level (mean ± SD across folds), the more exacting comparison; held-out values are single-split image-level estimates.

Aspect	Previous Pipeline [1]	This Work
Feature representation	EfficientNetB3, frozen; GAP + GMP (3072-d)	Seven backbones fine-tuned end-to-end
Best model	MLP over extracted features	DenseNet-121 (fine-tuned CNN)
Held-out accuracy	0.862	0.822
Held-out AUC	0.918	0.922
Held-out sensitivity	0.741	0.900
Held-out specificity	0.909	0.792
Animal-level CV AUC	0.681 ± 0.132	0.914 ± 0.019
Animal-level CV accuracy	0.624 ± 0.064	≈0.835 (mean ± SD of folds; Table 4)
Explainability	None (proposed only)	Grad-CAM reported
Model-comparison statistics	Logistic regression	DeLong + Nadeau–Bengio + Holm
Relative compute cost	Lower (frozen features)	Higher (end-to-end fine-tuning)

**Table 11:** Representative research using machine learning and thermal imaging to check for mastitis. Only each research’s actual performance is listed; “qualitative” refers to a study that reports detection capabilities but lacks a single headline indicator that is comparable in this context.

Study (Year)	Inputs	Method	Validation	Key Reported Result
Polat et al., 2010 [8]	IRT udder temperature	Se/Sp analysis vs. CMT/SCC	Herd	IRT detects subclinical mastitis (qualitative)
Coşkun & Aytekin 2021 [11]	IRT thermograms	Classification/regression tree	Herd	Early detection (qualitative)
Velasco-Bolaños 2021 [9]	IRT surface temperature	Correlation/threshold	Tropical-highland herd	Tracks subclinical mastitis (qualitative)
Bobbo et al., 2021 [13]	SCC/milk composition	LDA and other classifiers	Herd records	Accuracy 0.797, sensitivity 0.524
Santana et al., 2025 [12]	IRT + environment + production	XGBoost	14-month AMS data	AUC 0.843, high specificity
Pan et al., 2025 [14]	Electrical conductivity + SCC	SVM/feed-forward network	Cross-validation	SVM acc. 0.956, sens. 1.000; FNN AUC 0.981
Tian et al., 2020 [39]	EC + pH + milk yield	KNN/deep learning	Online	Clinical mastitis detection (qualitative)
This work	IRT thermal udder images	DenseNet-121 (fine-tuned CNN)	Nominal animal-level 5-fold	CV AUC 0.914 ± 0.019; held-out AUC 0.922; external accuracy 0.600 (*n* = 85, different farm)

## Data Availability

The data presented in this study are available on request from the corresponding author researchers seeking access to the image archive should contact the corresponding author to discuss a controlled-access arrangement.

## Supplementary Materials

The following supporting information can be downloaded at: https://www.mdpi.com/article/10.3390/vetsci13101031/s1, Table S1. Key technical specifications of the UNI-T UTx313 thermal imaging camera relevant to udder thermal-image acquisition; Table S2. Ingredients of the total mixed ration (TMR) and chemical composition on a dry-matter basis for lactating dairy cows in the biological-assessment group from Copenhagen and Delta Misr farms; Table S3. Held-out test performance with 95% confidence intervals widened by a conservative design-effect correction for unverified within-cow clustering (DEFF = 1 + (m − 1) × ICC, m = 2 images/cow, ICC = 0.7 shown as the most conservative bound; ICC = 0.3 and 0.5 are provided in the reproducibility repository); Table S4. Extended external-cohort validation metrics for DenseNet-121 against the CMT reference (*n* = 85 cows), computed on the continuous prediction score. The categorical metrics use the fixed 0.5 threshold carried over unchanged from internal validation; no threshold retuning was performed on the external cohort; Table S5. Quantitative comparison of DenseNet-121 Grad-CAM saliency against an automatically detected udder region, computed over the full 976-image archive. The udder region was estimated per image with an intensity-based method (Otsu thresholding, morphological cleanup, and center-weighted selection of the largest warm connected component, falling back to a fixed generous center-crop when no reliable region was found), then manually spot-checked on multiple random samples of 8–20 images before this analysis was run. “Peak activation” is defined as the top 20% of each image’s Grad-CAM values; Figure S1. ROC curves for image-level hold-out evaluation of the ten machine-learning models on the unaugmented test set. The curves represent corrected within-dataset performance and should not be interpreted as independent external validation; Figure S2. Calibration (reliability) plot for DenseNet-121 on the external cohort (*n* = 85), comparing mean predicted probability to observed CMT-positive fraction in five equal-sized bins. The dashed line indicates perfect calibration; Figure S3. Representative examples of the automatic udder-region detection used for Table S5 (*n* = 8 of 976). Top row: source thermal images with filename and reference label (H = healthy, M = mastitic). Bottom row: the detected region overlaid in red and its bounding crop box outlined in green. The leftmost pair illustrates the fixed-crop fallback used when no reliable warm-region separation is found (Section 4, Discussion); the remaining seven illustrate typical udder-localized detections.

## Abbreviations

The following abbreviations are used in this manuscript: AUC stands for area under the ROC curve; CNN for convolutional neural network; CV for cross-validation; EC for electrical conductivity; FNN for feed-forward neural network; IRT for infrared thermography; LDA for linear discriminant analysis; ROI for region of interest; SCC for somatic cell count; SCM for subclinical mastitis; Se for sensitivity; Sp for specificity; USST for udder skin surface temperature; and ViT for Vision Transformer. Because datasets, species, tagging, and validation procedures vary, reported metrics are as claimed by each source and are not necessarily directly comparable.

## References

[1] Elmasry A.S., Elwakeel E.A., Allam A.M., Attia M.F.A., Elmaria A.T., Badr E.E.M., Sallam S.M.A. (2026). Infrared Thermography and Machine Learning for Mastitis Detection in Dairy Cows: A Pilot Case Study in Egyptian Farms. Vet. Sci..

[2] El-Sayed A., Kamel M. (2021). Bovine Mastitis Prevention and Control in the Post-Antibiotic Era. Trop. Anim. Health Prod..

[3] Adkins P.R.F., Middleton J.R. (2018). Methods for Diagnosing Mastitis. Vet. Clin. N. Am. Food Anim. Pract..

[4] Duarte C.M., Freitas P.P., Bexiga R. (2015). Technological Advances in Bovine Mastitis Diagnosis: An Overview. J. Vet. Diagn. Investig..

[5] Lamanna M., Polizzi G., Bovo M., Cavallini D. (2026). Precision Livestock Farming for Dairy Cows. Encyclopedia of Livestock Medicine for Large Animal and Poultry Production.

[6] Lamanna M., Polizzi G., Vastolo A., Cavallini D. (2026). Milking Process and Milking Technology. Encyclopedia of Livestock Medicine for Large Animal and Poultry Production.

[7] Colak A., Polat B., Okumus Z., Kaya M., Yanmaz L.E., Hayirli A. (2008). Short Communication: Early Detection of Mastitis Using Infrared Thermography in Dairy Cows. J. Dairy Sci..

[8] Polat B., Colak A., Cengiz M., Yanmaz L.E., Oral H., Bastan A., Kaya S., Hayirli A. (2010). Sensitivity and Specificity of Infrared Thermography in Detection of Subclinical Mastitis in Dairy Cows. J. Dairy Sci..

[9] Velasco-Bolaños J., Ceballes-Serrano C.C., Velásquez-Mejía D., Riaño-Rojas J.C., Giraldo C.E., Carmona J.U., Ceballos-Márquez A. (2021). Application of Udder Surface Temperature by Infrared Thermography for Diagnosis of Subclinical Mastitis in Holstein Cows Located in Tropical Highlands. J. Dairy Sci..

[10] Usamentiaga R., Venegas P., Guerediaga J., Vega L., Molleda J., Bulnes F. (2014). Infrared Thermography for Temperature Measurement and Non-Destructive Testing. Sensors.

[11] Coşkun G., Aytekin İ. (2021). Early Detection of Mastitis by Using Infrared Thermography in Holstein-Friesian Dairy Cows via Classification and Regression Tree (CART) Analysis. Selçuk J. Agric. Food Sci..

[12] Santana R.C.M., Guimarães E.d.S., Caracuschanski F.D., Brassolatti L.C., da Silva M.L., Garcia A.R., Pezzopane J.R.M., Alves T.C., Tholon P., Santos M.V.D. (2025). Machine Learning Techniques Associated with Infrared Thermography to Optimize the Diagnosis of Bovine Subclinical Mastitis. Vet. Med. Int..

[13] Bobbo T., Biffani S., Taccioli C., Penasa M., Cassandro M. (2021). Comparison of Machine Learning Methods to Predict Udder Health Status Based on Somatic Cell Counts in Dairy Cows. Sci. Rep..

[14] Pan L., Chen X., Han D., Li N., Chen D., Wang J., Chen J., Huo X. (2025). Machine Learning-Based Clinical Mastitis Detection in Dairy Cows Using Milk Electrical Conductivity and Somatic Cell Count. Front. Vet. Sci..

[15] Zhang X., Kang X., Feng N., Liu G. (2020). Automatic Recognition of Dairy Cow Mastitis from Thermal Images by a Deep Learning Detector. Comput. Electron. Agric..

[16] Wang Y., Kang X., He Z., Feng Y., Liu G. (2022). Accurate Detection of Dairy Cow Mastitis with Deep Learning Technology: A New and Comprehensive Detection Method Based on Infrared Thermal Images. Animal.

[17] Zhang Q., Yang Y., Liu G., Ning Y., Li J. (2023). Dairy Cow Mastitis Detection by Thermal Infrared Images Based on CLE-UNet. Animals.

[18] Chu M., Li Q., Wang Y., Zeng X., Si Y., Liu G. (2023). Fusion of Udder Temperature and Size Features for the Automatic Detection of Dairy Cow Mastitis Using Deep Learning. Comput. Electron. Agric..

[19] Gayathri S.L., Bhakat M., Mohanty T.K., Chaturvedi K.K., Kumar R.R., Gupta A., Kumar S. (2024). Udder Thermogram-Based Deep Learning Approach for Mastitis Detection in Murrah Buffaloes. Comput. Electron. Agric..

[20] He K., Zhang X., Ren S., Sun J. Deep Residual Learning for Image Recognition. Proceedings of the IEEE Conference on Computer Vision and Pattern Recognition (CVPR).

[21] Huang G., Liu Z., van der Maaten L., Weinberger K.Q. Densely Connected Convolutional Networks. Proceedings of the IEEE CVPR.

[22] Tan M., Le Q.V. EfficientNet: Rethinking Model Scaling for Convolutional Neural Networks. Proceedings of the 36th International Conference on Machine Learning (ICML).

[23] Sandler M., Howard A., Zhu M., Zhmoginov A., Chen L.-C. MobileNetV2: Inverted Residuals and Linear Bottlenecks. Proceedings of the IEEE CVPR.

[24] Szegedy C., Vanhoucke V., Ioffe S., Shlens J., Wojna Z. Rethinking the Inception Architecture for Computer Vision. Proceedings of the IEEE CVPR.

[25] Dosovitskiy A., Beyer L., Kolesnikov A., Weissenborn D., Zhai X., Unterthiner T., Dehghani M., Minderer M., Heigold G., Gelly S. An Image Is Worth 16×16 Words: Transformers for Image Recognition at Scale. Proceedings of the International Conference on Learning Representations (ICLR).

[26] Liu Z., Lin Y., Cao Y., Hu H., Wei Y., Zhang Z., Lin S., Guo B. Swin Transformer: Hierarchical Vision Transformer Using Shifted Windows. Proceedings of the IEEE/CVF International Conference on Computer Vision (ICCV).

[27] Selvaraju R.R., Cogswell M., Das A., Vedantam R., Parikh D., Batra D. Grad-CAM: Visual Explanations from Deep Networks via Gradient-Based Localization. Proceedings of the IEEE ICCV.

[28] DeLong E.R., DeLong D.M., Clarke-Pearson D.L. (1988). Comparing the Areas under Two or More Correlated Receiver Operating Characteristic Curves: A Nonparametric Approach. Biometrics.

[29] Nadeau C., Bengio Y. (2003). Inference for the Generalization Error. Mach. Learn..

[30] Holm S. (1979). A Simple Sequentially Rejective Multiple Test Procedure. Scand. J. Stat..

[31] Zuiderveld K. (1994). Contrast Limited Adaptive Histogram Equalization. Graphics Gems IV.

[32] Vaswani A., Shazeer N., Parmar N., Uszkoreit J., Jones L., Gomez A.N., Kaiser Ł., Polosukhin I. Attention Is All You Need. Proceedings of the NeurIPS.

[33] Loshchilov I., Hutter F. Decoupled Weight Decay Regularization. Proceedings of the ICLR.

[34] Mann H.B., Whitney D.R. (1947). On a Test of Whether One of Two Random Variables Is Stochastically Larger than the Other. Ann. Math. Stat..

[35] Cohen J. (1960). A Coefficient of Agreement for Nominal Scales. Educ. Psychol. Meas..

[36] Saito T., Rehmsmeier M. (2015). The Precision-Recall Plot Is More Informative than the ROC Plot When Evaluating Binary Classifiers on Imbalanced Datasets. PLoS ONE.

[37] Magro S., Costa A., Cavallini D., Chiarin E., De Marchi M. (2024). Phenotypic variation of dairy cows’ hematic metabolites and feasibility of non-invasive monitoring of the metabolic status in the transition period. Front. Vet. Sci..

[38] Raghu M., Zhang C., Kleinberg J., Bengio S. Transfusion: Understanding Transfer Learning for Medical Imaging. Proceedings of the Advances in Neural Information Processing Systems (NeurIPS).

[39] Tian F., Wang Z., Yu S., Xiong B., Wang S. (2020). Clinical Mastitis Detection by On-Line Measurements of Milk Yield, Electrical Conductivity and Deep Learning. J. Phys. Conf. Ser..

