# Supplementary material for: Deep Learning and Explainable Artificial Intelligence for Infrared-Thermography-Based CMT-Status Classification in Dairy Cows: A Comparative Evaluation in Egyptian Farms
Source: Vet Sci. 2026 Sep 29;13(10):1031. doi: 10.3390/vetsci13101031 (PMC13709568; doi:10.3390/vetsci13101031)
Supplement: Supplementary file 1 [file vetsci-13-01031-s001.zip › vetsci-4522702-supplementary.pdf]

Supplementary Materials

Table S1. Key technical specifications of the UNI-T UTx313 thermal imaging camera relevant to udder thermal-image acquisition.

Parameter	Specification
**Camera model**	UNI-T UTx313
**Detector type**	UFPA, vanadium oxide
**IR resolution**	384 × 288 pixels
**Spectral range**	8–14 µm
**Pixel size**	12 µm
**Thermal sensitivity**	NETD ≤ 40 mK
**Color palettes**	Black hot, white hot, hottest, ironbow, rainbow

Table S2. Ingredients of the total mixed ration (TMR) and chemical composition on a dry-matter basis for lactating dairy cows in the biological-assessment group from Copenhagen and Delta Misr farms.

(a) Ingredients of the concentrate mixture, kg/head.

Copenhagen ingredient	kg/head	Delta Misr ingredient	kg/head
Corn silage	18	Corn	6.8
Alfalfa hay	6	Soyabean meal 46%	5.6
Wheat straw	0.2	Corn silage	32.9
Sugar beet pellets	3	Alfalfa hay	0
Yellow corn	4.5	Wheat bran	0
Potato starch dough	2	Wheat straw	1
Tomato dregs	6.5	Rice bran	1.5
Soybean meal	1	Molasses	0.5
Bypass soy	1	Water	0
Barley dregs	6	Liquid glycerin	0.1
Flaxseed	1	Limestone	0.092
Orange peel	6	Table salt	0.055
Sodium bicarbonate	0.1	Vitamex brand new	0.022
Calci Puff	0.16	Urea	0
Ekomos	0.01	Bicarbonate	0.280
Fixphen	0.015	Magnesium oxide	0.070
Dicalcium phosphate	0.07	Selenium	0.001
Mineral	0.04	Zinc	0.005
Vitamins	0.02	Vitafix (anti-toxin)	0.1
NaCl	0.12	Dicalcium phosphate	0.025
Sulfur	0.004	Protected fat (84%)	0.465

Copenhagen ingredient	kg/head	Delta Misr ingredient	kg/head
Bioplex Manganese	0.003		
Nitro-Tech	0.019		
Niacin	0.006		
Smart Amin	0.018		
Bioplex Zinc 20%	0.005		
Selenium	0.003		
Lesygim	0.03		
Liver Tonic	0.01		
Chelated copper	0.002		
Ye-Sacc yeast	0.005		

(b) Chemical composition on a dry-matter basis.

Parameter	Copenhagen	Delta Misr
Dry matter (%)	100%	100%
Crude protein (%)	15.02	16.13
NDF (%)	30.57	30.96
ADF (%)	20.07	15.64
Cellulose (%)	17.18	13.14
Hemicellulose (%)	10.49	15.32
Lignin (%)	2.89	2.5
NDICP (%)	2.23	1.87
ADICP (%)	1.03	1.18
Crude fat (%)	4.05	3.95
Ash (%)	9.34	9.86
NFC (%)	41.02	39.11
TDN (%)	71.77	71.51
DE1X (Mcal/kg DM) *	3.21	3.22
ME3X (Mcal/kg DM) *	2.56	2.57
ME4X (Mcal/kg DM) *	2.44	2.46
NEL3X (Mcal/kg DM) *	1.61	1.62
NEL4X (Mcal/kg DM) *	1.53	1.54

** If calcium soap of fatty acids has been included in the ration, it is not recovered in the crude fat value. Adjusted NDF: 28.34% on a DM basis. Adjusted NFC: 43.25% on a DM basis. Note: DM, dry matter; CP, crude protein; NDF, neutral detergent fiber; ADF, acid detergent fiber; TDN, total digestible nutrients; DE, digestible energy; ME, metabolizable energy; NEL, net energy for lactation.*

Table S3. Held-out test performance with 95% confidence intervals widened by a conservative design-effect correction for unverified within-cow clustering (DEFF = 1 + (m − 1) × ICC, m = 2 images/cow, ICC = 0.7 shown as the most conservative bound; ICC = 0.3 and 0.5 are provided in the reproducibility repository).

Model	Accuracy	AUC	Sensitivity	Specificity	Precision	F1
**ResNet-50**	0.741 (0.650–0.828)	0.879 (0.801–0.951)	0.903 (0.769–1.000)	0.679 (0.567–0.787)	0.515 (0.361–0.665)	0.654 (0.514–0.779)
**DenseNet-121**	0.822 (0.741–0.903)	0.923 (0.860–0.973)	0.901 (0.776–1.000)	0.791 (0.684–0.894)	0.620 (0.452–0.773)	0.733 (0.593–0.848)
**EfficientNet-B0**	0.807 (0.720–0.880)	0.910 (0.844–0.967)	0.922 (0.796–1.000)	0.763 (0.657–0.861)	0.595 (0.427–0.745)	0.721 (0.588–0.846)
**MobileNetV2**	0.727 (0.628–0.823)	0.860 (0.770–0.938)	0.830 (0.675–0.977)	0.688 (0.568–0.800)	0.501 (0.346–0.672)	0.623 (0.476–0.765)
**Inception-V3**	0.780 (0.684–0.862)	0.860 (0.774–0.929)	0.723 (0.529–0.903)	0.802 (0.695–0.895)	0.579 (0.403–0.755)	0.641 (0.473–0.781)
**ViT-B/16**	0.650 (0.552–0.758)	0.724 (0.611–0.835)	0.649 (0.455–0.839)	0.651 (0.528–0.767)	0.412 (0.266–0.574)	0.502 (0.348–0.655)
**Swin-Tiny**	0.635 (0.540–0.728)	0.715 (0.604–0.817)	0.724 (0.538–0.897)	0.602 (0.476–0.712)	0.407 (0.254–0.557)	0.518 (0.364–0.658)

Point estimates are unchanged from Table S1 (original bootstrap analysis, Table 3 main text); intervals are widened relative to the original analysis to account for the unverified pairing of left- and right-side images from the same cow. Full results across ICC = 0.3, 0.5, and 0.7 are available in the reproducibility repository (Section 2.10, Supplementary Data).

Table S4. Extended external-cohort validation metrics for DenseNet-121 against the CMT reference (n = 85 cows), computed on the continuous prediction score. The categorical metrics use the fixed 0.5 threshold carried over unchanged from internal validation; no threshold retuning was performed on the external cohort.

Metric	Value	95% bootstrap CI
Prevalence (mastitic)	40/85 (47.1%)	–
TN / FP / FN / TP @ 0.5 threshold	27 / 18 / 16 / 24	–
Accuracy	0.600	0.494–0.706
Sensitivity	0.600	0.449–0.750
Specificity	0.600	0.458–0.744
PPV	0.571	0.417–0.721
NPV	0.628	0.481–0.769
ROC-AUC	0.656	0.537–0.767
PR-AUC (baseline = prevalence = 0.471)	0.667	0.526–0.799
Calibration intercept (perfect = 0)	−0.184	–
Calibration slope (perfect = 1)	0.194	–

Table S5. Quantitative comparison of DenseNet-121 Grad-CAM saliency against an automatically detected udder region, computed over the full 976-image archive. The udder region was estimated per image with an intensity-based method (Otsu thresholding, morphological cleanup, and center-weighted selection of the largest warm connected component, falling back to a fixed generous center-crop when no reliable region was found), then manually spot-checked on multiple random samples of 8-20 images before this analysis was run. "Peak activation" is defined as the top 20% of each image's Grad-CAM values.

Metric	Value
Images analyzed (of 976; 0 skipped for no reliable mask)	976
Mean fraction of peak activation outside udder mask	0.453 (45.3%)
Median fraction of peak activation outside udder mask	0.432 (43.2%)
Mean fraction, correctly classified images	0.454 (45.4%)
Mean fraction, misclassified images	0.447 (44.7%)
Mean udder-mask coverage of frame (sanity check, n = 976)	0.50 (50%)
Images with implausible mask coverage (<10% or >70% of frame)	3 / 976 (0.3%)

Because the udder mask is an automatic intensity-based proxy rather than a verified anatomical segmentation, and because Grad-CAM's spatial resolution is limited by the final convolutional feature map, these figures should be read as an approximate, upper-bound estimate of background-linked attention rather than a precise measurement (Section 4, Discussion). The similarity between the correctly classified (45.4%) and misclassified (44.7%) subsets suggests this attention pattern is general rather than error-specific.

Figure S1. ROC curves for image-level hold-out evaluation of the ten machine-learning models on the unaugmented test set. The curves represent corrected within-dataset performance and should not be interpreted as independent external validation.

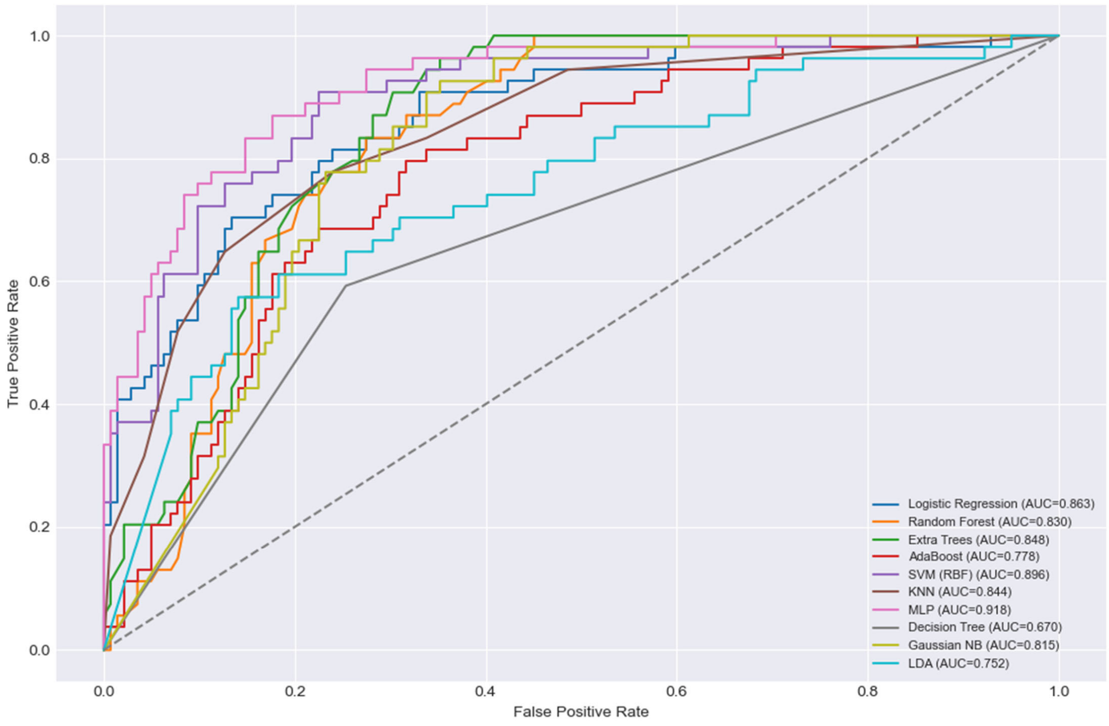

Figure S2. Calibration (reliability) plot for DenseNet-121 on the external cohort (n = 85), comparing mean predicted probability to observed CMT-positive fraction in five equal-sized bins. The dashed line indicates perfect calibration.

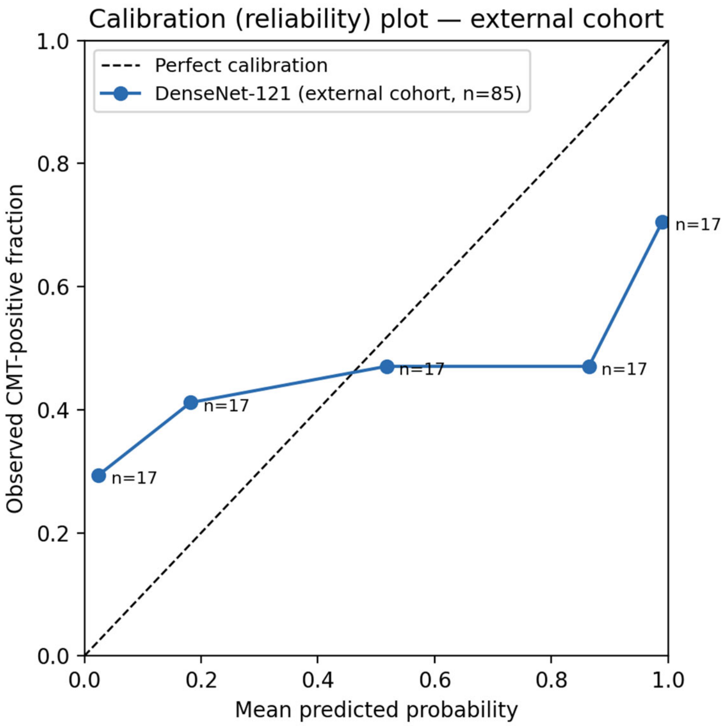

Figure S3. Representative examples of the automatic udder-region detection used for Table S5 (n = 8 of 976). Top row: source thermal images with filename and reference label (H = healthy, M = mastitic). Bottom row: the detected region overlaid in red and its bounding crop box outlined in green. The leftmost pair illustrates the fixed-crop fallback used when no reliable warm-region separation is found (Section 4, Discussion); the remaining seven illustrate typical udder-localized detections.

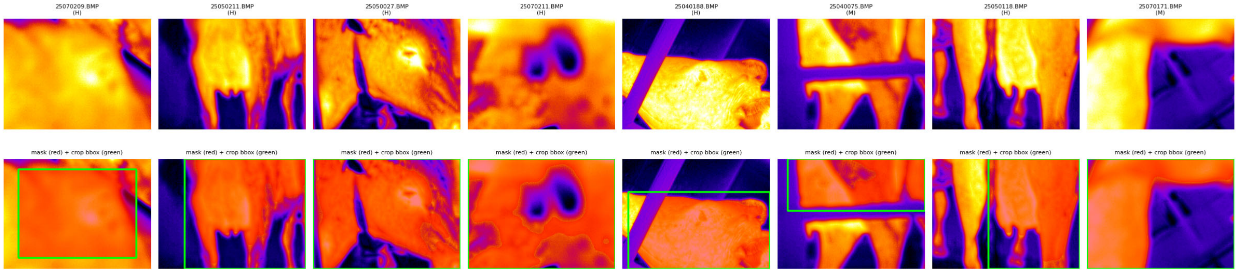

Sample drawn at random from the full 976-image archive; additional random samples of 8-20 images were reviewed during development and are not reproduced here. Detection method as described in the Table S5 caption.
